# Allosteric control of dynamin-related protein 1-catalyzed mitochondrial fission through a conserved disordered C-terminal Short Linear Motif

**DOI:** 10.21203/rs.3.rs-3161608/v1

**Published:** 2023-07-14

**Authors:** Isabel Pérez-Jover, Kristy Rochon, Di Hu, Pooja Madan Mohan, Isaac Santos-Perez, Julene Ormaetxea Gisasola, Juan Manuel Martinez Galvez, Jon Agirre, Xin Qi, Jason A. Mears, Anna V. Shnyrova, Rajesh Ramachandran

**Affiliations:** 1 Department of Biochemistry and Molecular Biology, University of the Basque Country, 48940 Leioa, Spain; 2 Instituto Biofisika, University of the Basque Country, 48940 Leioa, Spain; 3 Department of Pharmacology, Case Western Reserve University School of Medicine, Cleveland, OH 44106, USA; 4 Department of Physiology and Biophysics, Case Western Reserve University School of Medicine, Cleveland, OH 44106, USA; 5 Electron Microscopy and Crystallography Center for Cooperative Research in Biosciences (CIC bioGUNE), Bizkaia Science and Technology Park Bld 800, 48160-Derio, Bizkaia, Spain; 6 York Structural Biology Laboratory, Department of Chemistry, University of York, Heslington, YO10 5DD, York, UK; 7 Center for Mitochondrial Diseases, Case Western Reserve University School of Medicine, Cleveland, OH 44106, USA; 8 Cleveland Center for Membrane and Structural Biology, Case Western Reserve University School of Medicine, Cleveland, OH 44106, USA

**Keywords:** Dynamin-related protein 1, mitochondrial fission, intrinsically disordered domain

## Abstract

The mechanochemical GTPase dynamin-related protein 1 (Drp1) catalyzes mitochondrial fission, but the regulatory mechanisms remain ambiguous. Here we found that a conserved, intrinsically disordered, six-residue *S*hort *Li*near *M*otif at the extreme Drp1 C-terminus, named CT-SLiM, constitutes a critical allosteric site that controls Drp1 structure and function *in vitro* and *in vivo*. Extension of the CT-SLiM by non-native residues, or its interaction with the protein partner GIPC-1, constrains Drp1 subunit conformational dynamics, alters self-assembly properties, and limits cooperative GTP hydrolysis, leading to the fission of model membranes *in vitro*. *In vivo*, the availability of the native CT-SLiM is a requirement for productive mitochondrial fission, as both non-native extension and deletion of the CT-SLiM severely impair its progression. Thus, contrary to prevailing models, Drp1-catalyzed mitochondrial fission relies on allosteric communication mediated by the CT-SLiM, deceleration of GTPase activity, and coupled changes in subunit architecture and assembly-disassembly dynamics.

## Introduction

Intrinsically disordered proteins (IDPs) and structured proteins that contain intrinsically disordered regions (IDRs) are ubiquitous comprising nearly half of the human proteome^1–3^. In contrast to the relatively stationary loops and turns that connect secondary structure elements in compactly folded protein domains, IDRs persist as a highly dynamic conformational ensemble, which in many instances undergoes a localized disorder-to-order structural transition upon partner interactions (with protein, lipid, or nucleotide) and/or via various post-translational modifications (PTMs)^4–6^. Consequently, IDRs function as regulatory nodes or hubs that govern host protein function by transcribing biological information from multiple interactions and modifications into discernible alterations in local protein fold, dynamics, and macromolecular assembly, including protein condensation via liquid-liquid phase separation (LLPS)^7–10^.

**Figure 1.**
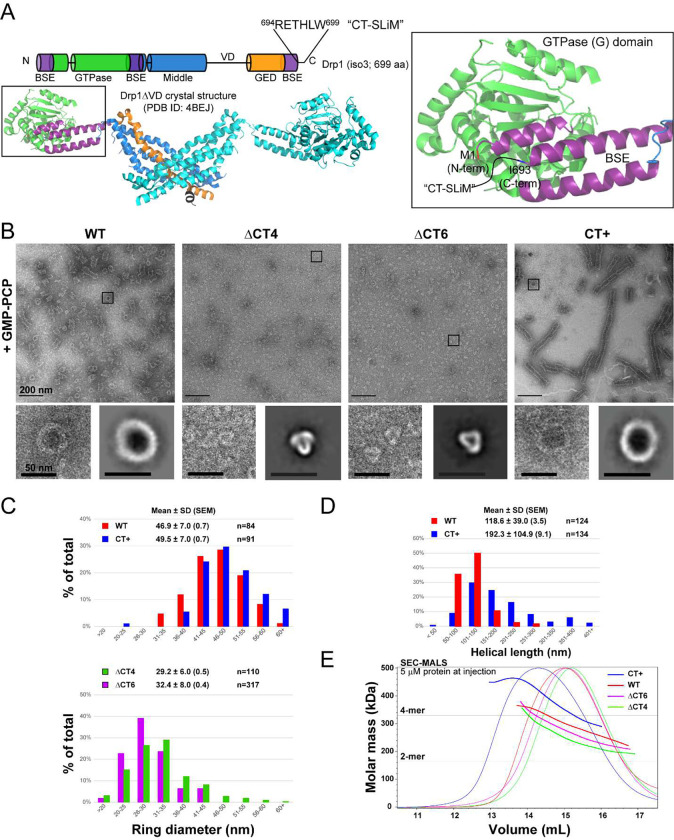
CT-SLiM modifications affect Drp1 oligomerization propensity and helical geometry. **(A)** (*Top*) Cartoon illustration of domain arrangement in the Drp1 primary structure. The location and polypeptide sequence of the CT-SLiM comprising C-terminal aa residues 694–699 in Drp1 isoform 3 is shown. (*Bottom*) Crystal structure of the Drp1DVD dimer with a corresponding color-coded representation of domain arrangement in a monomer. BSE is ‘bundle signaling element’, a three-helical bundle composed of helices derived from three discontinuous regions (purple), whereas the ‘stalk’ comprises a four-helical bundle composed of also discontinuous middle (blue; 3 helices) and GED (GTPase effector domain; orange; 1 helix) regions. GTPase (G) domain is shown in green. Connecting black lines represent a few prominent IDRs in Drp1. The VD connects the middle and GED regions, whereas the 80-loop and LIN loops are nested within the G and stalk (middle) domains, respectively. (*Inset*) A zoomed-in view of the BSE showing the well-resolved Drp1 N-terminal BSE helix (beginning from aa residue 1), whereas the last six residues of the Drp1 C-terminus (R694-W699), an IDR which we call the CT-SLiM and represented here by a curved black line, remain disordered. I693, the last resolved residue of the C-terminal BSE helix is highlighted. **(B)** Representative NS-EM images of WT Drp1 and CT variants in the presence of the non-hydrolyzable GTP analogue, GMP-PCP. Scale bar, 200 nm. *Insets* below each micrograph panel show zoomed-in views of the boxed regions in the above micrograph (left) and 2-D class averages of the predominant oligomer (ring) morphology (right). Insets scale bar, 50 nm. Data shown here are for mouse CT+ Drp1. Human CT+ Drp1 data are shown in Fig. S2A. **(C, D)** Histograms showing the distribution of assessed ring diameter (B) and helical polymer length (C) for WT Drp1 and CT variants. DCT4/6 strikingly do not form helical polymers. Mean ± SD (SEM) are indicated. ‘n’ refers to the number of particles. **(E)** SEC-MALS elution and molar mass profiles of human WT Drp1 and CT variants sieved through a Superose 6 10/300 GL column at 5 μM protein injection. Horizontal lines indicate the theoretical masses of a Drp1 dimer (2-mer) and tetramer (4-mer).

This generalized description of IDR structure and function also pertains to dynamin-related protein 1 (Drp1), a self-assembling, multi-domain GTPase that mechanochemically constricts tubular membrane intermediates *en route* to mitochondrial fission^11,12^. Drp1 contains multiple IDRs, ranging up to 134 amino acid (aa) residues in length, which make up >20% of its overall sequence^11,13,14^ (Fig. S1A). Yet, many of these IDRs are either absent or unresolved in any available Drp1 X-ray^13^ or cryo-EM structure^15,16^ to date (Fig. 1A and Fig. S1B) obscuring further functional characterization. These include: (i) *Mo*lecular *R*ecognition *F*eatures (MoRFs)^8,10^ of ~10–25 aa residues, such as MoRFs-1 and -2 embedded within the largely disordered variable domain (VD), enabling direct Drp1-membrane interactions^17^, and (ii) *S*hort *Li*near *M*otifs (SLiMs)^8,10^ of ~3–12 aa residues nested within highly structured domains, such as the G-domain ‘80-loop’ and stalk ‘L1N’ loop that direct protein-protein interactions during Drp1 self-assembly^14^. One such IDR is a unique stretch of ~6 aa residues at the Drp1 extreme C-terminus, which we call the CT-SLiM (Fig. 1A and Fig. S1A, B) that unlike other IDRs in Drp1 is remarkably highly conserved among metazoans (Fig. S1A). However, its function(s) remain largely unexplored.

Recent studies have indicated that this CT-SLiM constitutes an atypical PDZ domain binding motif (PBM) that specifically interacts with the PDZ domain-containing adaptor protein GIPC-1 (GAIP interacting protein, C-terminus 1)^18,19^. CT-SLiM-bound GIPC-1, in turn, associates with the F-actin minus-end-directed motor myosin VI (MYO6) to guide Drp1 presumably to F-actin-pre-constricted mitochondrial division sites^18–21^. However, whether or how direct GIPC-Drp1 interactions via the CT-SLiM influence Drp1 structure and/or function remains unknown.

Here, using a comprehensive toolkit of structural, cell biological, and *in vitro* reconstitution approaches, we show that a deletion (ΔCT) or a non-native extension (CT+) of the CT-SLiM distinctly alters Drp1 conformational dynamics, oligomerization propensity, self-assembly geometry, and cooperative GTPase activity, in addition to differentially affecting Drp1 capacity to remodel target membranes. We demonstrate that whereas the ΔCT variants exhibit a predictable loss-of-function by either altering or inhibiting membrane fission both *in vitro* and *in vivo*, the CT+ variants display an aberrant gain-of-function by robustly catalyzing membrane fission *in vitro*, while remaining repressed in mediating mitochondrial fission *in vivo*. By contrast, WT Drp1, which is limited to constricting membranes on its own *in vitro*, remarkably progresses toward membrane fission upon native CT-SLiM-effected GIPC-1 interactions.

Taken together, our data indicate a critical role for the native CT-SLiM in governing Drp1 structure, conformational dynamics, and mechanoenzymatic membrane remodeling activity. Furthermore, key partner protein interactions of Drp1, such as that of the CT-SLiM, emerge as an essential regulatory element in the allosteric control of Drp1 function during mitochondrial fission.

## Results

### CT-SLiM modifications alter Drp1 self-assembly propensity and geometry

To discern the role of the CT-SLiM, we generated a host of Drp1 variants with either truncated or extended C-termini (Fig. S1C). The truncated variants had the last four (ΔCT4) or six (ΔCT6) residues of the native CT-SLiM removed. In contrast, the extended variants (CT+) contained non-native sequences of different lengths and composition, including affinity and epitope tags, appended to the CT-SLiM. To enable purification, WT Drp1 and select CT variants were modified at the N-terminus with a His_6_ affinity tag (see Methods), which as previously shown^22,23^ did not affect Drp1 self-assembly or GTPase activity *in vitro*. Besides, N-terminally epitope (Myc)-tagged Drp1 effectively restored mitochondrial fission in Drp1 knockout (KO) cells^17,22^ indicating that these N-terminal modifications neither affect Drp1 function *in vivo*.

Negative-stain electron microscopy (NS-EM) analysis revealed significant alterations in the Drp1 oligomer structure due to the CT-SLiM modifications (Fig. 1B–D). In the presence of the non-hydrolyzable GTP analogue, GMP-PCP, which mimics GTP binding and promotes Drp1 helical self-assembly in solution^22^, WT Drp1 characteristically formed a mixture of oligomeric rings and higher-order spirals of a consistent diameter and length (Fig. 1B–D). In contrast, the ΔCT4 and ΔCT6 variants failed to assemble into any such regular higher-order structures. Instead, the ΔCT4 and ΔCT6 variants predominantly constituted triangularly shaped ‘nubs’ of much smaller dimensions with little to no indication of further higher-order self-assembly (Fig. 1B,C). Conversely, the CT+ variant formed consistently longer supramolecular helical assemblies, although similar in overall helical diameter to WT (Fig. 1B–D).

Size-exclusion chromatography-coupled multi-angle light scattering (SEC-MALS) analyses of these variants in the nucleotide-free *apo* state in solution revealed further differences in their oligomerization properties relative to WT (Fig. 1E). The ΔCT4 and ΔCT6 variants exhibited a sharp dimer-tetramer equilibrium similar to WT, albeit marginally tending toward minimal dimers under the conditions. In contrast, the extended CT+ variant largely favored higher-order oligomers consistent with its enhanced helical self-assembly in the presence of GMP-PCP (Fig. 1B and Fig. S2A, B). This greater oligomerization propensity of the CT+ variant relative to WT was evident over a wide range of protein concentrations (Fig. S2C). Besides, it was independent of the non-native CT+ sequence as this tendency was also manifest in a CT+* variant containing an extension of a different length (14 aa residues) and composition (Fig. S2D). Shortening the non-native CT sequence of the CT+ variant from 24 to 9 aa residues by proteolytic cleavage (CT+^sh^) significantly reduced its higher-order oligomerization propensity (Fig. S2E), although this remained noticeably greater than that of WT Drp1. On the other hand, shortening the N-terminal tag sequence from 36 to 7 aa residues had no palpable effect on Drp1 oligomerization (Fig. S2F).

These data indicated that the disordered Drp1 CT-SLiM is a critical determinant of Drp1 self-assembly and helical propagation.

### CT-SLiM modifications alter Drp1 conformational dynamics and structure

To gain insight into the molecular mechanisms underlying CT-SLiM function, we used NS-EM and performed 2-D image classification to assess the impact of the various CT modifications on Drp1 subunit conformational dynamics and self-assembly under different nucleotide-bound states (Fig. S3A).

**Figure 2.**
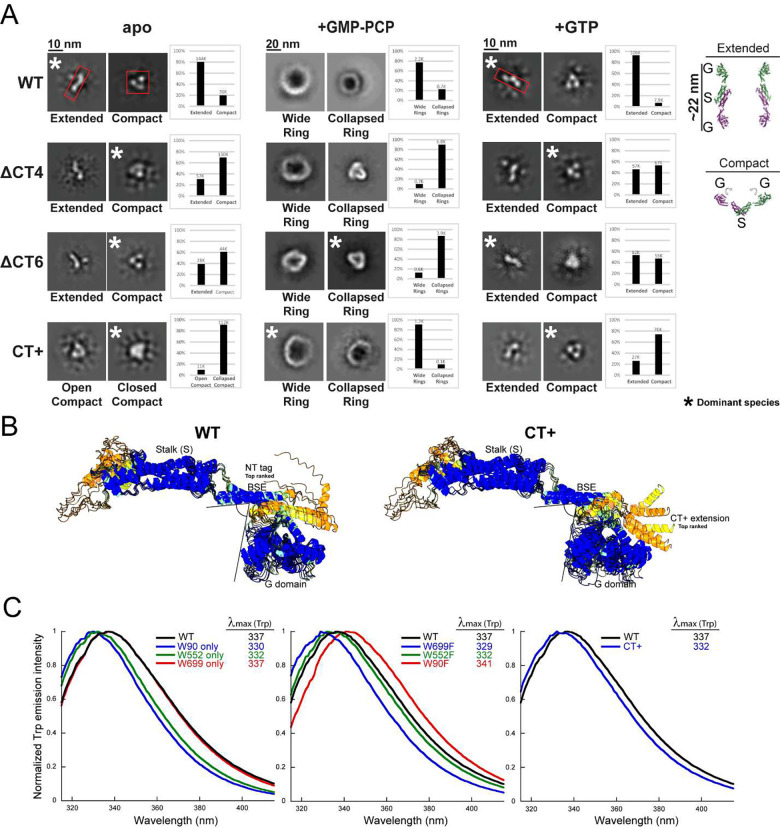
CT-SLiM modifications alter Drp1 conformational dynamics. **(A)** NS-EM 2-D class averages of dimers and oligomeric rings in the apo (left), GMP-PCP-bound (middle), and GTP hydrolysis (right) states for human WT Drp1 and CT variants. Drp1 dimer conformation in the apo and GTP hydrolysis states is classified as either ‘extended’ or ‘compact’, with the latter classified further into ‘open compact’ or ‘closed compact’ states, when both types are observed. Oligomeric ring morphology in the presence of GMP-PCP is classified into ‘wide’ and ‘collapsed’ ring states. Top-down and side-on views of the Drp1DVD dimer crystal structure (PDB ID: 4BEJ) are shown at the far right corner for ease of comparison with the NS-EM-detected Drp1 dimer morphologies. G refers to the G domain, whereas S refers to the stalk. **(B)** AlphaFold models of N-terminally tagged WT Drp1 and C-terminally tagged CT+ Drp1 following the AFDB colouring scheme, with blue representing high pLDDT and orange representing low pLDDT. The top ranked conformer is indicated near the corresponding affinity tags. Note that the non-native sequence extension in CT+ Drp1 propagates as an a-helix and juxtaposes with the G domain, causing a noticeable inward buckling of the G domain toward the BSE. The N-terminal tag, on the other hand, is mostly disordered and does not appear to contact the top of the G domain. **(C)** Normalized Trp emission spectra of human WT Drp1 in comparison to single W-only mutants (*left*), single W-to-F mutants (*middle*), and the CT+ Drp1 variant (*right*). The wavelength of maximum emission (λ_max_) is indicated.

In the *apo* state, we detected two different orientations for the WT Drp1 dimer — an ‘S-shaped’ top-down (or bottom-up) orientation and a ‘V-shaped’ side-on orientation with prominent densities evident for the dimeric stalk and the two individual GTPase (G) domains (Fig. 2A and Fig. S3B). Notably, in the S-shaped orientation, the G domains were set far apart, whereas in the V-shaped orientation, the G domains were positioned in close proximity. These orientations likely correspond to two different solution conformations of the Drp1 dimer — an S-shaped ‘extended’ conformer and an alternate V-shaped ‘compact’ conformer that possibly interconvert dynamically (Fig. 2A and Fig. S3C).

For the *apo* WT Drp1 dimer, the extended conformer was detected at a ~4-fold greater incidence than the compact conformer, indicating a greater residence time for the native dimer in the extended conformation (Fig. 2A). Remarkably, in striking contrast, the extended conformer was never found for the *apo* CT+ variant and was relatively poorly sampled by the ΔCT4/6 variants in the *apo* state (Fig. 2A). These data indicated that the native CT-SLiM restricts Drp1 conformational dynamics and retains Drp1 predominantly in the extended conformation, and that its absence or non-native extension in the ΔCT4/6 and CT+ variants relieves this auto-inhibition. Moreover, in the presence of GMP-PCP, the oligomeric rings formed by the ΔCT4/6 and CT+ variants were largely irregular or poorly ordered (Fig. 2A) suggesting that the native CT-SLiM also functions as a ‘spacer’ that sets the register and geometry of inter-subunit interactions during nucleotide-dependent helical self-assembly. Furthermore, unlike WT, which reverted to the extended dimer conformation upon GTP hydrolysis, the CT+ and ΔCT4/6 variants largely remained in the compact conformation (Fig. 2A and Fig. S3C). These data indicated that the compact CT+ Drp1 conformer likely mimics an ‘assembly-primed’ state based on its greater higher-order oligomerization propensity relative to WT both in absence and presence of nucleotide.

To understand the molecular basis of this putative CT+ gain-of-function, we used AlphaFold^24^ to predict the influence of the CT+ sequence extension on Drp1 structure (Fig. 2B). Remarkably, the computational data suggested that whereas the N-terminal His_6_ affinity tag in our WT Drp1 was mostly disordered, the non-native CT extension in CT+ Drp1 propagated as an α-helix in close apposition to the top of the G domain, potentially constraining dynamics at the adjacent nucleotide-sensitive G domain-BSE interface (Fig. 2B). Consistently, a direct comparison of the two structures both in isolation (Fig. 2B) and upon superposition into the available Drp1 polymer cryo-EM structure (Fig. S4A), revealed a slight inward buckling of the G domain toward the BSE in CT+ Drp1 compared to WT Drp1. In addition, given the proximity of the CT-SLiM to the stalk of the adjacent monomer in the Drp1 polymer (Fig. S4A), the modeling data further indicated that the CT+ extension may influence Drp1 subunit-subunit interactions during higher-order helical self-assembly.

We used *i*ntrinsic *T*ryptophan *F*luorescence *S*pectroscopy (iTFS)^25,26^ to experimentally validate these *in silico* predictions (Fig. 2C). Trp emission is highly sensitive to its microenvironment and therefore serves as an accurate probe of protein conformation or conformational changes^25,26^. When excited selectively at λ = 295 nm, the Trp emission spectrum is ‘blue-shifted’ (peaking at shorter wavelengths) when present in a nonpolar environment, and ‘red-shifted’ (peaking at longer wavelengths) when exposed to a polar or aqueous milieu. Drp1 contains three native Trp residues at positions 90, 552 and 699 (Fig. S1A). Of these, only W90 present in the G domain is structurally resolved^13^, whereas W552 and W699 are located in the disordered VD and CT-SLiM, respectively (Fig. S1A, S4B). Using site-directed Drp1 mutants that either retained one of the three native Trp or conversely contained only a single native Trp-to-Phe substitution, we ascertained that Drp1 Trp emission primarily originates from W699, the terminal residue of the CT-SLiM.

Consistent with the partial burial of W90 in the Drp1 G domain structure^13^ (Fig. S4B), the W90-only mutant displayed a pronounced blue shift in Trp emission relative to WT (Fig. 2C, *left* panel). Similarly, the W552-only mutant also exhibited a significant blue shift, albeit less than that of the W90-only mutant, indicating that W552 is also partially occluded from solvent in the VD conformational ensemble (Fig. 2C, *left* panel). By contrast, the W699-only mutant was pronouncedly red-shifted and was identical to WT in emission spectra (Fig. 2C, *left* panel). These data indicated that W699 in WT Drp1 is solvent accessible, and is the primary emitter largely owing to its location within Trp-Trp homo-FRET distance (~24 Å)^25^ of the high-energy FRET donor, W90 (Fig. S4B). Consistent with this assessment, the W90F mutant was substantially red-shifted (by 11 nm) compared to the W90-only mutant, whereas the W699F mutant was significantly blue-shifted (by 8 nm) relative to the W699-only mutant (Fig. 2C, *middle* panel). The W552F mutant, on the other hand, did not experience any such change (Fig. 2C, *middle* panel). These data confirmed that W699 in the WT Drp1 CT-SLiM is exposed to water and is highly responsive to its local environment. Notably, by contrast to WT Drp1, CT+ Drp1 emission was significantly blue-shifted (by 5 nm) indicating that W699 in CT+ Drp1 is instead buried and relatively solvent inaccessible (Fig. 2C, *right* panel). No such change in Trp emission was observed when the 36 aa-residue N-terminal His_6_ tag of WT Drp1 was replaced by a relatively short 7-aa residue overhang (Fig. S4C) indicating that the difference in the environment of CT-SLiM in CT+ Drp1 is primarily due to the non-native CT extension. Thus, together with the cryo-EM data and AlphaFold predictions, the iTFS data demonstrated that non-native CT extension of CT+ Drp1 alters CT-SLiM microenvironment and overall Drp1 conformation. WT and CT+ Drp1 thus populate distinct conformational states.

### CT-SLiM modifications differentially affect Drp1 cooperative GTP hydrolysis.

We next determined the impact of the various CT-SLiM modifications on Drp1 GTPase activity under assembly-restricted basal conditions in solution and upon unrestricted helical self-assembly on CL-containing membranes. Importantly, the CT variants retained the characteristic capacity of Drp1 to self-assemble on, and tubulate, CL-containing liposomes (Fig. S5). Furthermore, CT+ Drp1, similar to WT, assembled and disassembled on CL-containing, galactosylceramide-doped rigid lipid nanotubes (GalCer-NTs) in the presence of GMP-PCP and GTP, respectively (Fig. S6). These data indicated that the CT modifications did not affect stalk-mediated Drp1 interactions on membranes.

**Figure 3.**
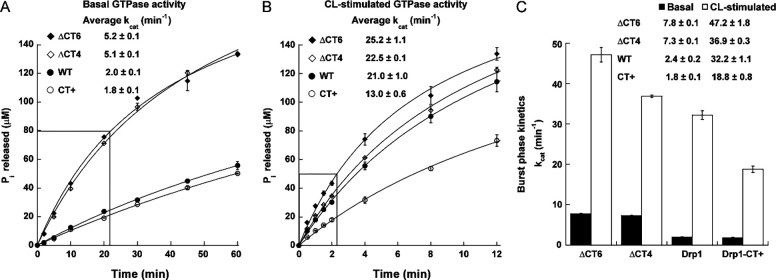
CT-SLiM modifications differentially affect Drp1 GTPase activity. **(A-C)** Basal and CL-stimulated GTPase activities of WT Drp1 and CT variants were measured using a malachite green-based colorimetric assay as described earlier. The concentration of inorganic phosphate (P_i_) released is plotted against time. In panels A and B, the average turnover number (k_cat_) from combined pre-steady state (burst phase; boxed regions) and steady-state kinetics is indicated. Panel C shows k_cat_ for the burst-phase only pre-steady state kinetics. Mean ± SEM are shown.

Surprisingly, however, the ΔCT4 and ΔCT6 variants both exhibited a ~3-fold greater rate of GTP hydrolysis in solution compared to the CT+ variant and WT, which were similar in basal GTPase activity (Fig. 3A). By contrast, the CT+ variant displayed a ~2-fold lower activity compared to the ΔCT4/6 variants and WT when assayed on CL-containing liposomes (Fig. 3B). Analysis of the pre-steady state ‘burst’ kinetics revealed that the ΔCT4/6 variants hydrolyzed GTP at a significantly faster rate than WT under both conditions (Fig. 3C). These data suggested that in the absence of the auto-inhibitory CT-SLiM, which restricts Drp1 conformational dynamics, transition state-dependent inter-subunit G-domain dimerization, cooperative GTP hydrolysis, GDP/Pi release, and G-domain dimer disassembly essential for successive rounds of GTP loading and hydrolysis are all markedly increased. Conversely, for the same reasons, in the presence of a non-native CT extension that artificially stabilizes inter-subunit Drp1 interactions and exaggeratedly promotes helical self-assembly as in CT+ Drp1, GTP turnover appears to be aptly decreased. Thus, fast dynamics in the absence of the CT-SLiM, and altered, slower dynamics in the presence of a non-native CT extension distinctively affect Drp1 cooperative GTPase activity relative to WT.

Together with its impact on Drp1 dimer structure, these functional data also raised the intriguing prospect that the native CT-SLiM functions as a ‘kinetic timer’ of Drp1’s GTP hydrolysis rate and coupled membrane remodeling activity.

### CT-SLiM controls Drp1-catalyzed membrane fission *in vitro*

We therefore addressed whether the differential GTPase activity, and altered conformational and self-assembly dynamics of the CT variants relative to WT translated to distinct membrane remodeling phenotypes. To this end, we tested the efficacy of our CT variants in directing the scission of suspended lipid nanotubes (NTs) mimicking the mitochondrial outer membrane at pre-constricted mitochondrial division sites. NTs ranging from tens to hundreds of nanometers in diameter were formed between polymer micropillars in a microfluidic chamber (see Methods). WT Drp1 and CT variants were then infused into this chamber in the presence of GTP while NT constriction and/or scission was monitored in real-time by fluorescence microscopy.

**Figure 4.**
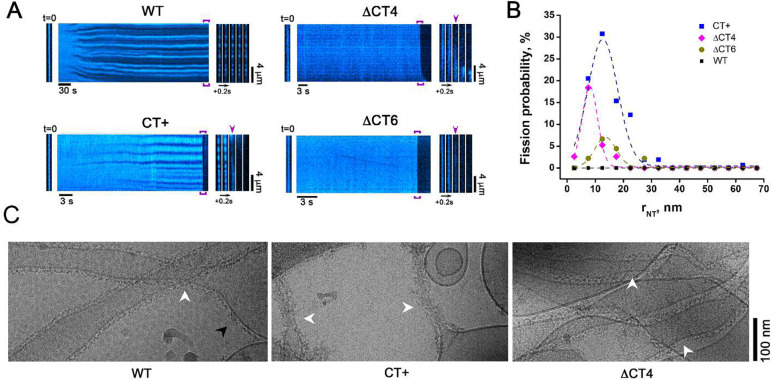
CT-SLiM modifications differentially affect membrane remodeling and fission. **(A)** Representative kymographs showing preformed NT remodeling upon addition of WT Drp1 and CT variants in the presence of 1 mM GTP. NT membrane fluorescence is displayed in cyan pseudocolor for clarity. *Left* images correspond to the initial frame of the kymographs. *Right* image sequences correspond to the framed region of the kymographs. Arrows indicate NT fission. **(B)** NT fission probability in the presence of GTP as function of NT initial radius upon addition of either 0.5 μM WT Drp1 (160 NTs, n = 3) or CT+ Drp1 (156 NTs, n = 3), or of either 2 μM DCT4 Drp1 (34 NTs, n = 3) or DCT6 Drp1 (38 NTs, n = 3). **(C)** Cryo-EM images showing WT Drp1 (*left*), CT+ Drp1 (*middle*), and DCT4 Drp1 (*right*) assembled on preformed NTs in the presence of 1 mM GTP. White arrowheads indicate Drp1 scaffolds on highly curved NT membranes. Black arrowhead in the *left* panel shows curvature-adaptable assembly of WT Drp1 also on relatively flat (low curvature) membrane regions, not observed with CT+ Drp1 (*middle panel*).

As previously shown^17,27^, WT Drp1 did not mediate NT fission on its own, but instead effectively constricted NTs to a final radius of 14 ± 2 nm independent of starting (initial) NT radii (Fig. 4A, Supplementary Movie 1). Surprisingly, however, both ΔCT4 and ΔCT6 Drp1 selectively mediated the fission of highly curved NTs, with the ΔCT4 variant exhibiting the greater fission efficiency of the two (Fig. 4A, 4B, Supplementary Movies 2, 3). Yet, for both of these variants, the area of membrane constriction prior to fission appeared to be highly limited and irresolvably narrow, being barely detected by fluorescence microscopy (Fig. 4A). These data indicated that a partial or complete deletion of the CT-SLiM severely impairs Drp1 self-assembly on membranes in the presence of GTP. This may be due to the greater GTP hydrolysis rate of the DCT4/6 variants (Fig. 3) causing rapid oligomer disassembly.

In stark contrast to the DCT4/6 deletion variants, the CT+ extension variants elicited a more robust constriction and fission of a broader range of initial NT radii ranging from ~5 to 35 nm (Fig. 4A, 4B, Supplementary Movie 2). Notably, the scission efficiencies of these variants directly corresponded to their higher-order oligomerization propensities with CT+ ≈ CT+* > CT+^sh^ (Fig. S7 and Fig. S2D, S2E). Thus, the markedly improved stability of the CT+ variants on membranes directly correlated with their robust membrane fission activities. Moreover, contrary to prevailing models, membrane fission activity was inversely correlated with the assembly-stimulated GTP hydrolysis rate on membranes, with CT+ variants of lower GTPase activity being more efficient in fission (Fig. S7). Remarkably yet, fission efficiency was directly proportional to the preponderance of the assembly-primed, compact dimer conformer in solution in the presence of GTP, sampled almost exclusively by the CT+ variants, but not WT (Fig. 2A).

To further assess the impact of the CT-SLiM modifications and imposed structural alterations on membrane remodeling, we used cryo-EM to analyze the self-assembly of WT Drp1 and CT variants on preformed membrane NTs in the constant presence of GTP (Fig. 4C). In agreement with the real-time fluorescence measurements, the cryo-EM data revealed that WT Drp1 formed organized helical polymers that constricted the NTs to a radius of ~15 nm. By contrast, CT+ Drp1 formed disorganized, ‘fuzzy coats’ that further constricted the membranes to critical radii of < 7 nm, frequently resulting in fission and consequent retraction of the cut NTs to the membrane reservoirs located on the EM grid. Interestingly, DCT4 Drp1 displayed helical polymers of highly variable diameter consistent with a near complete loss of CT-SLiM-imposed inter-subunit helical register and polymer geometry. Of note, under these conditions, WT Drp1 polymers were observed on both highly curved and relatively flat membrane regions, whereas the CT+ variant was curvature-selective with an acute preference for binding highly curved NTs (Fig. 4C).

Together, these data indicate that the CT-SLiM governs both Drp1 polymer geometry and dynamics on membranes, and that CT modifications differentially affect membrane curvature selectivity and fission activity.

### CT-SLiM interactions with GIPC-1 regulate Drp1-mediated membrane fission

Next, we determined how Drp1-GIPC-1 interactions via the CT-SLiM affected Drp1 structure, assembly, dynamics, and function.

GIPC-1 contains an N-terminal IDR in addition to a centrally located PDZ domain flanked by two unique GIPC homology domains (GH1 and GH2)^28^ (Fig. S8A). The GH1 and PDZ domains are involved in GIPC-1 multimerization^29^, whereas GH2 binds MYO6. In the absence of the N-terminal IDR and a PBM (PDZ ligand), GIPC-1 forms an auto-inhibited, PDZ-domain-swapped dimer that occludes both PBM and MYO6 binding sites^28^ (Fig. S8B). However, consistent with a previous report^29^, we found that in the unliganded state, full-length GIPC-1 exists in a fast dimer-monomer equilibrium largely favoring monomers (Fig. S8C–E). These data indicated that CT-SLiM binding may likely relieve GIPC-1 auto-inhibition, elicit GIPC-1 multimerization, and promote cooperative Drp1-GIPC-1 co-assembly. Consistent with this notion, multimeric GIPC-1 has previously been localized to membranes^29^ indicating a role for the GIPC multimerization in ligand protein (e.g. Drp1) confinement at target membrane sites.

**Figure 5.**
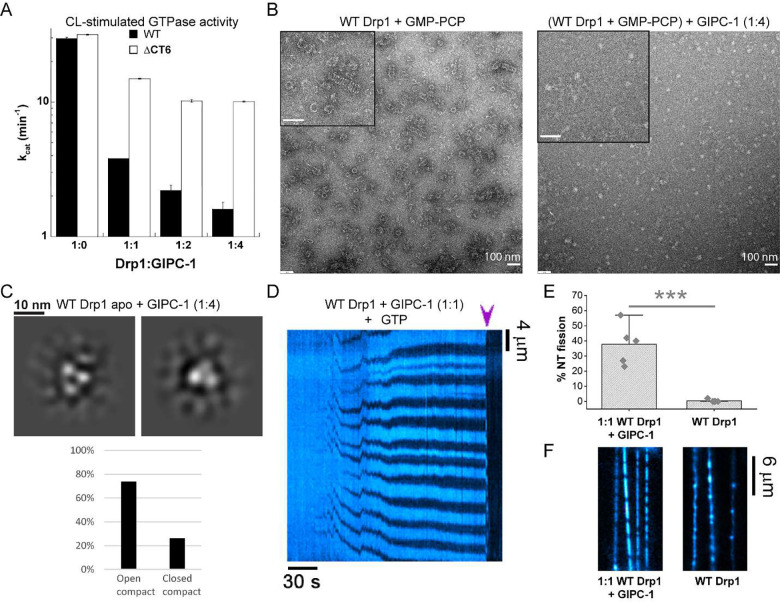
Native CT-SLiM interactions with GIPC-1 potentiate membrane fission *in vitro*. **(A)** CL-stimulated GTPase activities of WT and DCT6 Drp1 with increasing concentrations of GIPC-1. The average k_cat_ (mean ± SEM) is plotted versus Drp1:GIPC-1 molar ratio. **(B)** Representative NS-EM images of WT Drp1 incubated with GMP-PCP in the absence and presence of a 1:4 molar ratio of GIPC-1. *Inset* shows a magnified view. *Inset* scale bar, 100 nm. **(C)** NS-EM 2-D class averages of WT Drp1 in the *apo* state in the presence of a 1:4 molar ratio of GIPC-1. Only the compact conformers were observed for WT-Drp1 in the presence of GIPC-1. **(D)** Kymograph showing NT constriction and fission (arrowhead) by 2 μM WT Drp1 in the presence of GIPC-1 at a 1:1 molar ratio in the presence of 1 mM GTP. NT membrane fluorescence is displayed in cyan pseudocolor for clarity. **(E)** Percentage of NTs that underwent fission upon addition of either 2 μM WT Drp1 alone (45 NTs, 4 replicates), or a mixture of 2 μM each of WT Drp1 and GIPC-1 (49 NTs, 4 replicates), in the presence of 1 mM GTP is shown. Each point represents a replicate. Mean ± SD are shown. *** Statistically different at the 0.001 level. **(F)** Images showing the rigid polymerization of WT Drp1 alone versus the formation of much shorter scaffolds in the equimolar presence of GIPC-1 on NTs. Images shown were acquired approximately 2 minutes after the addition of the proteins at 2 μM final concentration each in the presence of 1 mM GTP. RhPE channel is shown. Pseudocolor is used for clarity.

GIPC-1 robustly inhibited the assembly-stimulated GTPase activity of WT Drp1 on CL-containing membranes in a concentration-dependent manner (Fig. 5A). Control experiments with the ΔCT6 Drp1 variant, by contrast, showed a relatively modest inhibition, nonetheless indicating the presence of additional GIPC-1 binding sites besides the CT-SLiM (Fig. 5A). In NS-EM experiments, GIPC-1 potently inhibited the GMP-PCP-induced self-assembly of WT Drp1 into rings and helices in solution, indicating that GIPC-1 binding prevents the CT-SLiM-mediated helical propagation of Drp1 (Fig. 5B). Consistent with the presence of additional binding sites, GIPC-1 also inhibited the GMP-PCP-induced formation of triangular nubs by ΔCT6 Drp1 (Fig. S9A).

Similarly, GIPC-1 potently inhibited WT Drp1-mediated tubulation of CL-containing liposomes (Fig. S9B). ΔCT6 Drp1 membrane remodeling activity, on the other hand, was not comparably affected (Fig. S9C). Interestingly, under these conditions, at an equimolar ratio of WT Drp1 and GIPC-1, amorphous assemblies of Drp1 and GIPC-1 were readily observed in solution, whereas at much higher ratios (1:4), linear and bundled filaments of assembled protein, reminiscent of Drp1 copolymerization with another adaptor, namely mitochondrial dynamics protein of 49 kDa or MiD49^30^, were evident (Fig. S9B). Together, these data indicated that GIPC-1 interactions via the CT-SLiM alters Drp1 self-assembly geometry, with pronounced effects on membrane remodeling as determined by the lack of ordered helical self-assembly and resultant membrane tubulation.

Further, NS-EM 2-D classification of *apo* WT Drp1 dimers in the presence of GIPC-1 in solution revealed that the exclusive presence of the assembly-primed, compact Drp1 conformer (Fig. 5C), in contrast to the auto-inhibited, extended Drp1 conformer observed in GIPC-1’s absence (Fig. 2A). Of note, additional density representing GIPC-1 was not readily evident from our 2-D class averages reflecting either a dynamic interaction of GIPC-1 with WT Drp1 in the *apo* state, or a substantial overlap of GIPC-1 density with the closely spaced domains of the compact WT Drp1 conformer. In the case of ΔCT6 Drp1, however, various extra densities and altered subunit arrangements were observed attesting to the presence of additional GIPC-1 binding sites (Fig. S9D).

Remarkably, in the NT fission assay, when co-assembled with GIPC-1, WT Drp1 assembled into scaffolds that constricted the NTs, recurrently leading to complete membrane scission (Fig. 5D, 5E, Supplementary Movie 5). Notably, at the relatively high protein concentration (2 μM each) used for this experiment, WT Drp1 alone, in the absence of GIPC-1, rapidly polymerized into rigid scaffolds, rendering kinks in the NTs (Fig. 5F) and precluding membrane fission. However, in the presence of GIPC-1, the protein scaffolds grew more measuredly in a manner similar to that of CT+ Drp1 prior to fission (Fig. 4A, Supplementary Movie 2). Thus, GIPC-1 association with the CT-SLiM appeared to disengage Drp1 inter-subunit interactions that promote extensive Drp1 polymerization but restrict local membrane constriction. Furthermore, the decreased assembly-stimulated GTPase activity observed in the presence of GIPC-1 seemed to enable the potent membrane remodeling leading to fission. Thus, WT-Drp1 in the presence of GIPC-1 mimics CT+ Drp1, which on its own exhibits reduced GTPase activity and altered CT-SLiM interactions.

### CT-SLiM interactions are critical for Drp1-catalyzed mitochondrial fission *in vivo*

**Figure 6.**
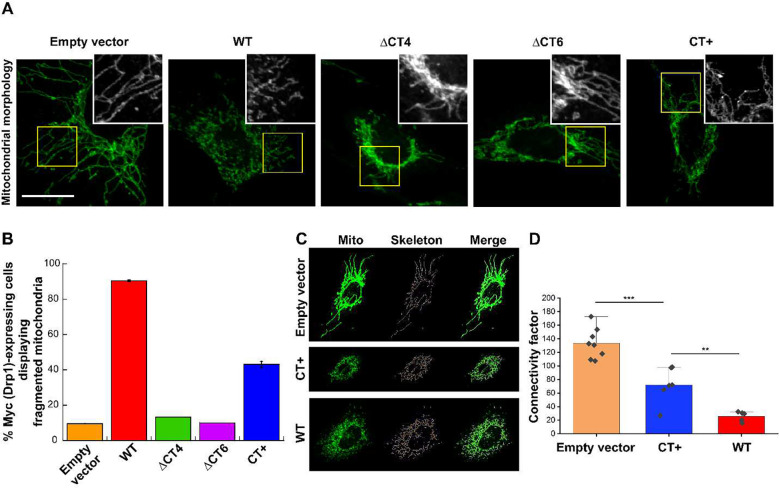
Native CT-SLiM interactions are essential for mitochondrial fission *in vivo*. **(A)** Representative images of mitochondrial morphology in Drp1 KO MEFs expressing Myc-tagged WT Drp1 or CT variants. Boxed areas (yellow) are zoomed and shown in grayscale in *insets*. Individual and merged image color panels are shown in Figs. S10 and S11. **(B)** Percentage of Myc (Drp1)-expressing cells displaying fragmented mitochondria are plotted for WT Drp1 versus CT variants. Total number (n) of Myc (Drp1)-expressing cells analyzed for each variant is n = 91 (WT Drp1), n = 92 (CT+ Drp1), n = 17 (DCT4 Drp1), n = 31 (DCT6 Drp1), and n = 70 (empty vector). Mean ± SD are shown. **(C)** Representative images of mitochondrial morphology and corresponding skeletons used for network connectivity analysis. **(D)** Analysis of network connectivity as in panel C. Connectivity factor is defined as total network length (pixels) per number of separate skeletons detected using the ImageJ skeletonize plugin. Each point represents a cell. Mean ± SD are shown. *** Statistically different at the 0.001 level; ** Statistically different at the 0.01 level (unpaired *t*-test, equal variance not assumed).

To determine whether the CT-SLiM modifications correspondingly affect Drp1-catalyzed mitochondrial fission *in vivo*, we examined and compared mitochondrial morphology in Drp1 KO mouse embryonic fibroblasts (MEFs) overexpressing either N-terminally Myc-tagged WT, ΔCT4, or the ΔCT6 variant, or the C-terminally Myc/FLAG-tagged CT+ variant (Fig. 6 and Fig. S10, S11A–C). Empty vector-transfected Drp1 KO MEFs displayed extensively hyperfused mitochondria owing to unopposed mitochondrial fusion in absence of Drp1-catalyzed mitochondrial fission (Fig. 6 and Fig. S10). As expected, exogenous Myc-WT Drp1 overexpression effectively rescued and restored mitochondrial fragmentation (Fig. 6 and Fig. S10). Nevertheless, consistent with previous studies, overexpression of the ΔCT4 and ΔCT6 variants had no palpable effect, with the great majority of cells displaying a pronounced perinuclear clustering of hyperfused mitochondria (Fig. 6 and Fig. S10). These data reiterated that interactions mediated by the CT-SLiM are essential for Drp1 function *in vivo*. Surprisingly, however, the CT+ variant, despite containing the native CT-SLiM, was also manifestly impaired in restoring mitochondrial fission (Fig. 6. and Fig. S10, S11). Thus, in spite of its apparent gain-of-function in effecting model membrane fission *in vitro*, the CT+ variant is nevertheless perturbed in effecting partner protein interactions, either with self, and/or with adaptors such as GIPC-1, essential for mitochondrial fission *in vivo*.

From the collective data, we conclude that the native CT-SLiM is a critical structural and functional determinant of physiologically relevant Drp1-catalyzed mitochondrial fission, and that its perturbations influence Drp1 function both *in vitro* and *in vivo*.

## Discussion

Structural and functional plasticity are two interlinked characteristics of IDRs^10,31,32^. This is best exemplified by the longest and best-recognized IDR in Drp1, the VD, which is involved in multiple protein-protein and protein-lipid interactions via various identified MoRFs and SLiMs^14^. Remarkably, the VD is auto-inhibitory to premature Drp1 self-assembly in solution^14,33,34^, while conversely promoting Drp1 self-assembly and function upon partner interactions, specifically with target lipids on mitochondrial membranes^14,17,22,35^, thus reflecting the VD’s duality and functional diversity. However, the VD and various other IDRs in Drp1 (e.g. the 80-loop) are relatively poorly conserved (Fig. S1A) and are subject to extensive tissue- and organism-specific alternative splicing^36^, indicating that some of their ascribed functions may not be entirely universal. Here, we demonstrate that the contrastingly highly conserved CT-SLiM, previously implicated in Drp1 transport^18^, is yet another critical, multifunctional ‘toggle’ that not only governs Drp1 conformational stability and dynamics, but also further directs Drp1 self-assembly, assembly geometry, and cooperative GTP hydrolysis to facilitate partner protein-guided membrane constriction and fission.

We demonstrate that the native CT-SLiM is an essential intra- and inter-molecular interaction motif that not only governs Drp1 subunit conformational dynamics and oligomerization propensity, but also functions as a ‘spacer’ that directs Drp1 self-assembly and propagation in the proper helical register. In addition, the CT-SLiM also functions as an auto-inhibitory motif, which in the absence of alleviating binding partners such as GIPC-1, restricts high membrane curvature generation (superconstriction) in order to control and enable partner protein-regulated membrane fission. Based on its composition (^694^RETHLW^699^), we surmise that a combination of electrostatic and hydrophobic interactions mediated by its highly conserved N- (via R^694^ and E^695^) and C-termini (via L^698^ and W^699^), respectively, facilitates this critical role. Interestingly, T^696^ nested within this motif is thought to be a potential site for phosphorylation by Ser/Thr kinases^18,37^ already known to modify the disordered VD at various locations^38–40^ to regulate Drp1 function.

Modifications of the CT-SLiM, either by truncation or non-native extension, differentially alter these functions not only by conformationally restricting Drp1 dynamics in solution, but also by affecting the curvature-adaptability of the Drp1 polymer on membranes essential for the massive constriction of large diameter tubular membrane intermediates during mitochondrial fission. Consistently, CT+ Drp1, which promotes stabilizing but promiscuous self-assembly interactions with neighboring subunits, and ΔCT4/6 Drp1, which spuriously form out-of-register polymers, though both assembly-competent, are rendered acutely curvature-sensitive in catalyzing membrane fission *in vitro*, while remaining inhibited in mediating physiologically relevant mitochondrial fission *in vivo*.

Remarkably, the Drp1 CT-SLiM, though structurally disparate, functionally parallels the much longer yet disordered C-terminal proline-rich domain (PRD) of prototypical dynamin in its regulatory capacity^41,42^, albeit in distinctive ways. Like the CT-SLiM, the PRD binds partner proteins essential for dynamin-catalyzed membrane fission *in vivo*^43,44^. Similar to that of the Drp1 CT-SLiM (ΔCT4/6), deletion of the PRD in dynamin (ΔPRD) results in increased GTPase activity^14^. These data indicate that the unpartnered CT-SLiM, much like the PRD functions as a *negative regulator* of inter-subunit G domain-dimerization necessary for cooperative GTPase activity. Correspondingly, CT+ Drp1, containing a ‘mini-PRD’-like non-native extension, duplicates dynamin^45,46^ in mediating the fission of model membranes independent of protein partners or receptors *in vitro*^45,46^, a phenomenon not evident with WT Drp1 in our assay setup under our experimental conditions^17^. These data suggest that an effector-induced dampening of the GTP hydrolysis rate and/or increased residence time of the GTP- or transition state-bound Drp1 oligomer on the membrane, instead of robust GTP hydrolysis and rapid oligomer disassembly, potentiates membrane-remodeling leading to complete membrane fission. In this regard, Drp1 conserves the characteristic feature of typical small molecular weight signaling GTPases (e.g. Ras, Rho), which reside alternatively in the GTP-bound, functional “on” and post-GTP hydrolysis GDP-bound, quiescent “off” states, interconverted by GEFs and GAPs, respectively^47,48^. For Drp1, target receptors and lipids likely fulfill these roles. Recent studies show that the extreme C-termini of the distantly related atlastins^49,50^, involved in ER membrane fusion, function in a similar auto-regulatory capacity^50,51^. Thus, from an evolutionary standpoint, the extreme C-terminus may represent a critical, conserved, regulatory feature of all dynamin superfamily proteins (DSPs)^49^.

Our combined experimental and theoretical modeling data further reveal that the non-native CT+ extension, by virtue of its spurious intra- and inter-molecular interactions, strongly restricts the conformational dynamics of the minimal Drp1 dimer in solution prior to higher-order self-assembly, in addition to artificially increasing oligomer stability and order in presence of GMP-PCP, or upon self-assembly on membranes. Furthermore, given the proximity of the native CT-SLiM to the nucleotide-responsive, dynamically swiveling BSE-stalk interface of an adjacent dimeric subunit in the Drp1 oligomer^15^, the non-native CT+ extension likely restricts GTP hydrolysis-dependent BSE-stalk interfacial movements responsible for oligomer disassembly. The non-native CT+ extension may thus conformationally prime Drp1 for dynamics-resistant oligomerization, consequently enhancing its membrane remodeling capacity, albeit artificially. For WT Drp1, binding partners GIPC-1, which binds the native CT-SLiM^18^ and thus restricts BSE-stalk conformational motion, and MiD49/51, which directly binds the BSE-stalk interface^15,30^ and alters assembly geometry, likely enable this conformational priming reaction essential for mitochondrial fission. Interestingly, GIPC-1^DN-IDR^ is a domain-swapped dimer, whose longitudinal dimension and inter-PDZ domain spacing (~10 nm) closely approximates the distance between the two G domains in the V-shaped compact conformer of the Drp1 dimer^19^. By dynamically bridging the two G domains via CT-SLiM interactions, GIPC-1 may function to steer the Drp1 dimer toward the assembly-primed compact conformation. Other contextual partners, such as mitochondrial fission factor (Mff), fission factor 1 (Fis1), and MiD49/51^52^, may play similar independent and/or synergistic roles in controlling this Drp1 conformational equilibrium between ‘auto-inhibited’ and ‘assembly-primed’ states as previously alluded^16,53^.

Our data further indicate that Drp1-GIPC-1 interaction(s) via the auto-inhibitory native CT-SLiM primes Drp1 for greater membrane remodeling by potentiating the force-generating conformational rearrangements necessary for catalyzing membrane fission. We surmise that GIPC-1 does so by displacing the CT-SLiM and alleviating the curvature-restrictive Drp1-Drp1 interactions that the CT-SLiM mediates to enable membrane superconstriction, and ultimately, fission. The CT+ variants, prematurely relieved of this CT-SLiM-mediated auto-inhibition due to promiscuous interactions of their non-native extensions, mimic this conformational transition even in the absence of GIPC-1 *in vitro*. Thus, akin to other Drp1 IDRs^14^, the CT-SLiM constitutes yet another critical regulatory node that controls Drp1 structure and function via partner protein-guided conformational transitions to accomplish mitochondrial fission.

Finally, we note that the triangular nubs formed by the ΔCT4 and ΔCT6 variants in presence of GMP-PCP in solution are highly reminiscent of the triangular arrangement of WT Drp1 dimers in presence of GMP-PCP and MiD49 observed in the aforementioned cryo-EM study^15^. Similarly, the linear fibrils of Drp1 observed in the presence of excess GIPC-1 are evocative of the linear copolymers of Drp1 and MiD49^30^ formed under similar conditions. These observations reaffirm our notion that partner protein interactions steer and direct Drp1 inter-subunit spacing, oligomerization geometry, nucleotide-sensitive conformational rearrangements, and assembly-disassembly dynamics, and are thus indispensable for physiologically relevant Drp1-catalyzed mitochondrial fission.

In summary, our data demonstrate that the native, disordered CT-SLiM motif is an essential structural and functional determinant of Drp1 function in mitochondrial division.

## Methods

### Protein production

Human Drp1 (isoform 3) WT, ΔCT4, and ΔCT6 subcloned in pRSET C (Invitrogen), CT+ subcloned in pET21b (Novagen), and CT+* subcloned in pET Biotin His_6_ FLASH (Addgene Plasmid #30184) were expressed and purified using a combination of His_6_-affinity and ion exchange chromatography as previously described^22,54^. Mouse CT+ identical to human CT+ in length (699 aa residues) and also in composition except for eight alternative residues within the G domain (3) and VD (5) was obtained from Addgene (Plasmid # 72927)^55^ and produced using the same protocol. The CT+ variant was referred to as Drp1-C in our previous study^27^. CT+^sh^ was produced from mouse CT+ by human rhinovirus (HRV) 3C protease cleavage. For iTFS measurements, single Trp-only mutations (two of the three native Trp mutated to Phe) and single Trp-to-Phe mutations (one of the three native Trp mutated to Phe) were introduced by site-directed mutagenesis in human WT Drp1 subcloned in pRSET C. A non-native Trp present in the N-terminal His_6_ affinity tag of pRSET C was substituted with Phe in the pertinent constructs used in iTFS experiments. WT Drp1 with a short 7-aa residue N-terminal tag derived from HRV 3C protease digestion was obtained from the Mears lab and is described elsewhere^56^. For the studies in Drp1 KO MEFs^57^, human Drp1 WT, ΔCT4, and ΔCT6 were subcloned in pCMV-Myc (Clontech) and expressed with an N-terminal c-Myc epitope tag, whereas human CT+ was subcloned in pCMV6 (Origene), which conversely appended tandem c-Myc and FLAG epitope tags at the C-terminus. GST-tagged mouse GIPC-1 subcloned in pGEX-6P1 (Cytiva) was expressed and purified using standard protocols. The N-terminal GST tag was removed post-purification by HRV3C proteolysis. Protein aggregates formed during GIPC-1 production as previously noted^28^ were removed by high-speed centrifugation at 20,000xg for 30 min at 4°C and/or by gel filtration over a Bio-Rad SEC650 column at 4°C. All proteins were stored in buffer A (20 mM HEPES, pH 7.5, 150 mM KCl) containing 1 mM DTT and 10% (v/v) glycerol.

### Liposome and GalCer-NT production

All lipids were obtained from Avanti Polar Lipids Inc. Liposomes containing 25 mol% bovine heart cardiolipin (CL), 35 mol% dioleoylphosphatidylethanolamine (PE), and 40 mol% dioleoylphosphatidylcholine (PC) were prepared in buffer A by extrusion through 400-nm pore-diameter polycarbonate membranes and used in NS-EM and CL-stimulated GTPase assays as described earlier^22^. Rigid lipid NTs composed of 25 mol% CL, 35 mol% PE, and 40 mol% C24:1 β-D-galactosylceramide (GalCer) were prepared using a sonication protocol as described elsewhere^58^, and used for negative-stain EM experiments.

### SEC-MALS

SEC-MALS analysis was performed as previously described^22^. Briefly, WT Drp1 and CT variants at the indicated injection concentrations were sieved through a Superose 6 10/300GL column attached to an ÄKTApure FPLC system (Cytiva) connected in line with DAWN Heleos-II 18-angle MALS and Optilab T-rEX differential refractive index (dRI) detectors from Wyatt Technology. Full-length GIPC-1 (10 μM at injection) was sieved using a Superdex 200 10/300 GL column similarly. Data were analyzed using the ASTRA 7 software from Wyatt Technology.

### NS-EM and data processing

Negative-stain samples were prepared using 2% (w/v) uranyl acetate (Polysciences, Inc.) on carbon-coated grids as previously described.

For the analysis of oligomeric ring structures in the presence of GMP-PCP, 2 μM Drp1 was incubated with 1 mM GMP-PCP in buffer A containing 2 mM MgCl_2_ and 1 mM DTT final for 30 minutes. Samples were imaged on a Tecnai T12 (FEI Co.) electron microscope at 120 keV, and 10–15 images were acquired using a Gatan 4k × 4k camera at a magnification of 49,000x. For analysis of dimer single particles classes, the apo (Drp1 at 2 μM), +GTP (1 mM), and +GIPC (8 μM) samples were imaged on a TF-20 FEG electron microscope (FEI Co.) operating at 200 kV and recorded at 50,000x magnification with a Tvips Tietz 4k × 4k CMOS-based camera to collect 200 micrographs for each condition.

Data processing was done in cryoSPARC. CTF correction was done using Patch CTF. For all conditions, ~100 particles were manually selected to create an initial picking template for automated picking. For each unique 2D class average identified, individual templates were selected, and all samples were searched using those templates to determine if those class averages were also represented in these samples, even if it was not the predominant class average. The GMP-PCP samples’ initial particle stacks were 10,000–20,000 particles, after one round of classification, while final stacks were 1500–6000 particles. The single particle samples’ initial particle stacks were 300,000–400,000 and required several rounds of 2D classification (2–4 iterations) to sort through the low SNR associated with small particles. Final stacks were 55,000–180,000 particles.

For negative-stain imaging of Drp1 alone on membranes, Drp1 (2 μM final) was incubated with 25% CL-containing liposomes (50 μM final total lipid) for 30 minutes in buffer A containing 1 mM DTT. For experiments with GIPC-1, Drp1 (1.5 μM) was pre-incubated with GIPC-1 (6 μM) for 15 min at room temperature before addition of liposomes (50 μM final total lipid) and incubation for an additional 15 min.

Membrane tube and oligomeric ring diameters were determined as previously described^59^. Briefly, a broad sampling of rings/helices/tubes were imaged throughout the grid. Measurements were made in ImageJ (NIH) and distributions were generated using either Microsoft Excel or Graphpad Prism.

### Computational 3-D model prediction

The atomic models of the different Drp1 constructs in their monomeric forms were calculated using AlphaFold version 2.1.1^60^ running on the Viking Cluster (University of York), using templates from PDB structures with date of deposition up to 14 May 2020. Multiple Sequence Alignments (MSA) were run on the full sequence databases (‘--db_preset=full_dbs’). 8 CPUs and two CUDA-enabled Graphics Processing Units (GPU) were used for each job. Five models were produced by default for each construct; the one with highest average pLDDT was taken. The compatibility of the produced models with a helical arrangement was tested and illustrated by superposing the constructs onto the cryo-EM structure of human Drp1 (PDB ID: 5WP9, EMDB map EMD-8874) using the GESAMT software^61^ of the CCP4 suite^62^. The calculated AlphaFold models showed varying degrees of predicted accuracy (average ± SD): 82.46 ± 10.37 for C-terminally tagged CT+ Drp1 and 64.24 ± 21.51 for N-terminally tagged WT Drp1 in which the tag remained mostly disordered.

### iTFS

iTFS spectral measurements were performed in a 4 × 4 mm quartz cuvette (Starna Cells, Inc., Atascadero, CA) at 25°C using a Fluorolog 3–22 photon-counting spectrofluorometer (Horiba Jobin Yvon) equipped with a 450-W xenon lamp, double-excitation and double-emission monochromators, a cooled PMT housing, and a temperature-controlled cuvette compartment. WT Drp1, CT+, and site-directed mutants were diluted to 0.5 μM final in buffer A containing 1 mM DTT. Buffer background- and instrument-corrected Trp fluorescence spectra were obtained by selectively exciting Trp at 295 nm (2-nm bandpass) and emission monitored at 1-nm increments between 315 and 415 nm (2-nm bandpass). Data are averages of three scans for each sample.

### NT fission assay

Fluorescently labeled lipid NTs were made as previously described^63^. Briefly, 40 μm silica beads covered by hydrated membrane lamella of the desired membrane composition (PC:PE:CL:RhPE 55:29.5:15:0.5 mol%) were mechanically rolled on top of a SU8 micropillar array manufactured on the cover slip surface of a microfluidic chamber. The chamber was initially perfused with buffer A containing 2 mM MgCl_2_, 1 mM EDTA, 1 mM DTT, and 0.5 mM n-propyl gallate final. Upon rolling of the beads, small membrane reservoirs formed on top of the pillars were interconnected by freely suspended NTs. The protein(s) of interest was/were perfused into the microfluidic chamber in the presence or absence of 1 mM GTP final in the same buffer. Drp1 and GIPC-1 were premixed at an equimolar ratio immediately before perfusion into the chamber.

NT remodeling and fission were monitored using an inverted fluorescence microscope (Nikon Eclipse Ti, Japan) equipped with a 100X/1.49NA objective lens, a CoolLed pE-4000 light source at a low (10%) intensity, and a Zyla 4.2 sCMOS camera (Andor, Ireland). μManager software was used for image acquisition. Image processing (background subtraction and kymograph building) and statistical analysis were performed using Fiji package in ImageJ^64^ and OriginPro 8.0 software, respectively.

### Cryo-EM of Drp1 on preformed NTs

The NTs were produced directly on the glow-discharged Quantifoil R 2/2 300 mesh copper grid. A 2 μL drop of buffer B (10 mM HEPES, pH 7.5, 150 mM KCl) containing 1 mM MgCl_2_ and 1 mM GTP was placed on the grid. NTs were formed by rolling lamella-covered silica beads as described above for NT formation on micropillars^63^. The resulting NTs were attached to the edges of the holes in the Quantifoil film. Upon NT stabilization, 2 μL of the protein of interest was added to the NTs-containing grid (final protein concentration 0.5 μM). Upon 5 min incubation of the NTs with protein, the excess liquid was removed by blotting with an absorbent filter paper on both sides of the grid for 2 seconds, using a Vitrobot system (Thermofisher) set at 18°C and 90% humidity. Subsequently, the sample was abruptly vitrified by plunging into liquid ethane (−184 °C). The vitrified grids were maintained in liquid nitrogen and visualized on a JEOL JEM2200FS/CR microscope equipped with a field emission gun operated at 200 kV and an in-column Ω energy filter. During imaging, non-tilted, zero-loss 2D images were recorded under low-dose conditions, utilizing the ‘Minimum Dose System (MDS)’ of Jeol software, with a total dose on the order of 30–40 electrons/Å^2^ per exposure and at defocus values ranging from 1.5 to 4.0 *μ*m. The microscope’s in-column Omega energy filter helped us record images with an improved signal-to-noise ratio (SNR) by zero-loss filtering, using an energy-selecting slit width of 20 eV centered at the zero-loss peak of the energy spectra. Digital images were recorded in linear mode on a 3840 × 3712 (5 *μ*m pixels) Gatan K2 Summit direct detection camera (Gatan Inc.) using DigitalMicrograph^™^ (Gatan Inc.) software, at nominal magnifications of 2000× and 25000×, with a pixel size of 1.6 nm and 0.154 nm respectively. Images were subsequently treated and analyzed using Fiji software^64^.

### Cell biology and immunostaining

Drp1 KO MEFs were plated on 0.1% gelatin (G1393, Sigma) coated coverslips in 12-well plates. 24 hr after plating, the cells were transfected with the vectors expressing the indicated Myc-tagged Drp1 constructs by using the *Trans*IT^®^-2020 transfection reagent (MIR 5404, Mirus). 24 hr after transfection, the cells were fixed with 4% PFA in PBS for 20 min at room temperature before 5 min permeabilization with 0.1% Triton-X-100. Cells were then incubated with blocking buffer (5% normal goat serum (31872, Thermofisher Scientific), 0.05% Triton-X-100 in PBS) for 1 hr at room temperature. Primary antibodies against Myc (Drp1) and Tom20 (mitochondria) were incubated overnight at 4°C. The secondary Alexa-488-labeled goat anti-rabbit (A11034, ThermoFisher Scientific) and Alexa-568-labeled goat anti-mouse (A11031, ThermoFisher Scientific) antibodies were incubated for 1 hr at room temperature. The expression of Myc-tagged Drp1 and mitochondrial morphology were examined by using anti-Myc (sc-40, Santa Cruz), and anti-Tom20 (11802-1-AP, Proteintech) antibodies, respectively. Confocal images were obtained using a 60X oil-immersion objective mounted on an Olympus Fluoview 1000 or 3000 confocal microscope and analyzed by Fiji-ImageJ (NIH).

### Quantification of mitochondrial connectivity

Images of cells expressing Myc (Drp1) were selected based on the plot profile maximum of the Alexa Fluor 568 (red) channel, set to 120 ± 20 a.u. For cells expressing the empty vector, the selection was based on Alexa Fluor 488 (green) intensity, with the maximum in 100 ± 0 a.u. The selected images were analyzed with an in-house Fiji^64^ macro consisting in four main steps: i) background subtraction, ii) binary mask creation, iii) skeletonize plugin^65^ and iv) analyze skeletons command. The resulting dataset was further analyzed using OriginPro software. Branch length was used to calculate the total network length and the average branch length. For the empty vector, all branches below 4 pixels were excluded from the analysis. For the number of skeletons, all skeletons below 4 pixels were deleted for the empty vector. All skeletons with length of 0 pixels were excluded from analysis. The connectivity factor was obtained by dividing the number of skeletons by the total network length.

## Supplementary Material

Supplement 1
