# Supplementary material for: Allosteric control of dynamin-related protein 1-catalyzed mitochondrial fission through a conserved disordered C-terminal Short Linear Motif
Source: Res Sq. 2023 Jul 18:rs.3.rs-3161608. Originally published 2023 Jul 14. Preprint. [Version 2] doi: 10.21203/rs.3.rs-3161608/v2 (PMC10371074; doi:10.21203/rs.3.rs-3161608/v2)
Supplement: Supplement 1 [file NIHPPrs3161608v2-supplement-1.pdf]

Supplementary figure legends

Figure S1. CT-SLiM conservation in metazoans, its location at a critical inter-subunit interface in the helical oligomer, and the various CT-SLiM modifications used in the study. **(A)** Clustal Omega (1.2.4) multiple sequence alignment of human Drp1 (from the ubiquitously expressed and shortest 699 aa isoform 3 used in the study; NCBI Reference Sequence: NP_005681.2) with that of mouse (*Mus musculus*; NP_001021118.1), frog (*Xenopus laevis*; NP_001080183.1), zebrafish (*Danio rerio*; AAH55521.1), Fruitfly (*Drosophila melanogaster*; NP_608694.2, and nematode (*Caenorhabditis elegans*; AAD49861.1). Sequences are color-coded in correspondence with the cartoon illustration of the Drp1 primary structure above. The location of the various prominent IDRs are indicated above. **(B)** Top-down views of the Drp1ΔVD tetramer (PDB ID: 4BEJ; left) and of the full-length Drp1 oligomer in association with the soluble domain of adaptor MiD49 (PDB ID: 5WP9; right) showing the location of the CT-SLiM. In the oligomer structure, the resolved CT portion is located adjacent to the BSE-stalk interface of the neighboring subunit as indicated by arrows. **(C)** Polypeptide sequences of the various CT modifications used in this study. See Methods for additional details.

Figure S2. NS-EM and SEC-MALS analysis of CT variants. **(A)** Representative NS-EM images of human WT Drp1 and the CT+ Drp1 variant in the presence of the non-hydrolyzable GTP analogue, GMP-PCP. Scale bar, 200 nm. *Insets* show 2-D class averages of the predominant oligomer (ring) morphology. *Insets* scale bar, 50 nm. **(B)** Histograms showing the distribution of assessed helical polymer length for WT Drp1 versus human CT+ Drp1. **(C-F)** Comparison of SEC-MALS profiles of human WT Drp1 containing a 36 aa residue N-terminal His_6 tag with (C) CT+ Drp1 (mouse) at 10 μM, 5 μM, and 2.5 μM injection concentrations, (D) with human WT Drp1 containing a much shorter 7 aa residue tag at the N-terminus, (E) with CT+ Drp1 (mouse) and a N-terminally His_6 tagged CT+* Drp1 (human), and (F) with CT+ Drp1 (mouse) and $CT+^{sh}$ Drp1 containing a much shorter 9 aa residue CT extension. See Fig. S1C and Methods for additional details. For panels D-F, the protein concentration at injection is indicated.

Figure S3. NS-EM 2-D classification workflow and analysis of CT variants under different states. **(A)** Workflow of NS-EM 2-D classification. See Methods for further details. **(B)** Analysis of dimer conformation for human WT Drp1 and CT variants in the *apo* and GTP hydrolysis states from NS-EM micrographs. Scale bar, 70 nm. The diameter of the circles is 40 nm. *Insets* show the predominant 2-D class averages. *Inset* scale bar, 10 nm. (C) Close-up views of WT Drp1 and CT+ Drp1 dimers (2-D class averages) in the apo and GTP hydrolysis states in solution. The compact conformation of CT+ Drp1 versus the extended conformation of WT Drp1 likely results from a pronounced inward buckling of the two G domains toward the central stalk in CT+ Drp1 as shown on the right. G is G-domain and S is stalk.

Figure S4. AlphaFold modeling and iTFS reveal distinct conformational alterations in CT+ Drp1. **(A)** Superposition of the AlphaFold-derived monomer structures for WT Drp1 and CT+ Drp1 on the Drp1 oligomer cryo-EM structure (PDB ID: 5WP9). One monomer in the cryo-EM structure is replaced by an AFDB scheme-colored AlphaFold structure. Note that the G domain in CT+ Drp1 is more pronouncedly buckled toward the G domain of the neighboring subunit on its left relative to WT Drp1 (denoted by the shrinking of a double-headed arrow placed in between

the two G domains). *Insets* show zoomed-in views of the altered conformation and bucking of the CT+ Drp1 G domain relative to WT Drp1. **(B)** Surface and ribbon representations of the G domain and BSE showing the proximity of W90 (red) in the G domain to the last resolved residue (I693, blue) in the BSE C-terminal helix (magenta). Distance between W90 and I693 was calculated to be 17.9 Å, within Trp-Trp homo-FRET distance (~24 Å) to adjacent but unresolved W699. (C) Normalized Trp emission spectra of two human WT Drp1 constructs containing a 36 aa-residue versus a 7 aa-residue N-terminal tag. λ_{max} indicated above shows no effect of tag length and sequence on Trp emission intensity originating from W699 in the CT-SLiM.

Figure S5. All CT variants retain membrane remodeling capacity. (A) NS-EM of WT Drp1 and CT variants on CL-containing liposomes. Scale bar, 200 nm. **(B)** Histograms showing the distribution of membrane tube diameters for WT Drp1 and the CT variants.

Figure S6. WT and CT+ Drp1 are both capable of assembly and disassembly on preformed membrane tubes. NS-EM of WT Drp1 and CT+ Drp1 assembled on rigid GalCer-NT in the *apo* state (top row) and upon addition of GMP-PCP (middle row) and GTP (bottom row). Scale bar, 50 nm. Boxed regions are zoomed and shown as *insets*.

Figure S7. Increased NT fission correlates with suppression of GTPase activity. (A) NT fission activity of the CT+ variants compared to WT Drp1. n.s., not significant. **(B)** Relative CL-stimulated GTPase activities of the CT+ variants shown as a percentage of the maximum exhibited by WT Drp1.

Figure S8. Structural and biochemical characterization of full-length GIPC-1. (A) Cartoon representation of domain arrangement in the GIPC-1 primary structure, and a corresponding color-coded AlphaFold model of the full-length GIPC-1 monomer. **(B)** Crystal structure of the PDZ domain-swapped GIPC-$1^{\Delta N51}$ dimer (PDB ID: 5V6B). One of the two monomers is color-coded in correspondence to the structures shown in panel A. Top and side views are shown. Distance between the target motif (PBM) binding sites of the two PDZ domains was calculated to be ~94.2 Å. **(C)** SDS-PAGE of full-length GIPC-1 after GST excision. **(D)** SEC-MALS analysis of purified, full-length GIPC-1 injected at 10 μM and sieved using a Superdex 200 10/300 GL column. Molar masses corresponding to monomer (1-mer) and dimer (2-mer) states are indicated by horizontal lines. An intermediate mass reveals a fast monomer-dimer equilibrium. **(E)** NS-EM of full-length GIPC-1 at 8 μM protein concentration. Homogeneously discoid profiles averaging ~19 nm in diameter were observed, but could not be further resolved by 2-D class averaging.

Figure S9. GIPC-1 imposes an alternate assembly geometry on Drp1. (A) Representative NS-EM images of ΔCT6 Drp1 incubated with GMP-PCP in the absence and presence of a 1:4 molar ratio of GIPC-1. Scale bar, 100 nm. **(B, C)** NS-EM of WT Drp1 (C) and ΔCT6 Drp1 (D) assembled on CL-containing liposomes in the absence (left) and presence of 1:1 (middle) and 1:4 (right) molar ratio of GIPC-1. For WT Drp1, membrane tubulation was inhibited in the presence of 1:1 GIPC-1. Instead, amorphous protein assemblies (black arrowhead) in solution were observed. At the 1:4 ratio, however, linear (black arrow heads) and bundled protein filaments (white arrowhead) likely representing hybrid copolymers of Drp1 and GIPC-1 were observed. Neither Drp1 nor GIPC-1 alone formed such entities at the concentrations used. For ΔCT6 Drp1, which cannot bind

GIPC-1 using the native CT-SLiM, membrane tubulation was largely preserved, although the tubes approached relatively narrow diameters in the presence of GIPC-1. **(D)** NS-EM 2-D class averages of ΔCT6 Drp1 in the *apo* state in the presence of a 1:4 molar ratio of GIPC-1. In contrast to WT Drp1, various other conformations besides the compact dimer, including extended chains and paired dimers were observed indicating the presence of additional, likely non-specific binding sites for GIPC-1.

Figure S10. CT-SLiM modifications impair mitochondrial fission *in vivo*. (A) Drp1 KO MEFs overexpressing Myc-tagged WT Drp1 and CT variants were immunostained using anti-Myc and anti-Tom20 (mitochondria) antibodies. Representative images from each group are shown. Uncropped panels for Drp1 (red), mitochondria (green), and merge are displayed along with an enlarged panel of the boxed region. Scale bar (uncropped full-size images), 50 μm. Scale bar (enlarged images), 20 μm.

Figure S11. CT+ Drp1 is defective in mitochondrial fission *in vivo*. (A) Same as in Fig. S10, but with CT+ Drp1 probed instead by an anti-FLAG antibody. CT+ Drp1 contains tandem Myc and FLAG tags as part of its non-native CT extension. See Methods for details. **(B)** Total lysates were harvested from Drp1 KO MEFs (B) and HEK293T cells (C) overexpressing either empty vector, Myc-tagged WT Drp1, or combined Myc/FLAG-tagged CT+ Drp1, and analyzed by western blotting using the indicated antibodies. In the case of CT+ Drp1, when expressed in both Drp1 KO MEFs and HEK293T cells, the downstream FLAG tag was more accessible to antibody staining than the upstream Myc tag positioned close to the CT-SLiM. Representative blots are shown.

Supplementary movies legends

Movie S1: NT constriction upon addition of 0.5 μM WT Drp1 in the presence of 1 mM GTP, corresponding to the kymograph in in Fig. 4A. RhPE fluorescence is shown.

Movie S2: NT constriction and fission upon addition of 0.5 μM CT+ Drp1 in the presence of 1 mM GTP, corresponding to the kymograph in in Fig. 4A. RhPE fluorescence is shown.

Movie S3: NT constriction and fission upon addition of 2 μM ΔCT4 Drp1 in the presence of 1 mM GTP, corresponding to the kymograph in in Fig. 4A. RhPE fluorescence is shown.

Movie S4: NT constriction and fission upon addition of 2 μM by ΔCT6 Drp1 in the presence of 1 mM GTP, corresponding to the kymograph in in Fig. 4A. RhPE fluorescence is shown.

Movie S5: NT constriction and fission upon addition of equimolar concentrations (2 μM each) of WT Drp1 and GIPC-1, corresponding to the kymograph in in Fig. 5D. RhPE fluorescence is shown.

Figure S1

A

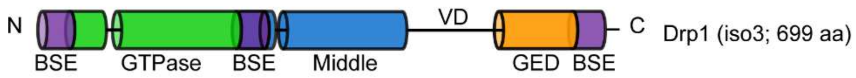

GTPase (G) domain; Middle domain (Stalk); Variable domain (VD)/IDR *contd. next page*

```
                 BSE (N-term helix)
Human       MEALIPVINKLQDVFNTVGA--DIIQLPQIVVVGTQSSGKSSVLESLVGRDLLPRGTGIV 58
Mouse       MEALIPVINKLQDVFNTVGA--DIIQLPQIVVVGTQSSGKSSVLESLVGRDLLPRGTGVV
Frog        MEALIPVINKLQDVFNTVGS--DVIQLPQIVVVGTQSSGKSSVLESLVGRDLLPRGTGVV
Zebrafish   MEALIPVINKLQDVFNTVGA--DIIQLPQIAVVGTQSSGKSSVLESLVGRDLLPRGTGIV
Fruitfly    MEALIPVINKLQDVFNTVGS--DSIQLPQIVVLGSQSSGKSSVIESVVGRSFLPRGTGIV
Nematode    MENLIPVVNKLQDVFATLGRKEDQIQLPQIVVVGSQSAGKSSVLENLVGRDFLPRGTGIV
            ** ****:******* *:*    * ******.*:*:**:*****:*.:***.:******:*
                         80-loop (IDR)
Human       TRRPLILQLVHVSQEDKRKTTGEENGVEAEEWGKFLHTKNKLYTDFDEIRQEIENETERI 118
Mouse       TRRPLILQLVHVSPEDKRKTTGEENGVEAEEWGKFLHTKNKLYTDFDEIRQEIENETERI
Frog        TRRPLILQLVHVSSDDRRKTSGDENGVEADEWGKFLHTKNKIYTDFDEIRQEIENETERI
Zebrafish   TRRPLILQLVHVDPEDRRKT-SEENGVDGEEWGKFLHTKNKIYTDFDEIRQEIENETERV
Fruitfly    TRRPLVLQLIYSPLDDRENRSAENGTSNAEEWGRFLHTK-KCFTDFDEIRKEIENETERA
Nematode    TRRPLILQLNHVALDDESKRRRSNGTLLTDDWAMFEHTGSKVFTDFDAVRKEIEDETDRV
            *****:*** :   :*. :   .:.    ::*. * **  * :****  :*:***:**:*

Human       SGNNKGVSPEPIHLKIFSPNVVNLTLVDLPGMTKVPVGDQPKDIELQIRELILRFISNPN 178
Mouse       SGNNKGVSPEPIHLKVFSPNVVNLTLVDLPGMTKVPVGDQPKDIELQIRELILRFISNPN
Frog        SGNNKGISSEPIHLKIFSPNVVNLTLVDLPGMTKVPVGDQPKDIEIQIRELILRYISNPN
Zebrafish   SGNNKGISDEPIHLKIFSPHVVNLTLVDLPGITKVPVGDQPKDIELQIRELILKYISNPN
Fruitfly    AGSNKGICPEPINLKIFSTHVVNLTLVDLPGITKVPVGDQPEDIEAQIKELVLKYIENPN
Nematode    TGVNKGISLLPISLKIYSHRVVSLSLVDLPGITKIPVGDQPVNIEEQIREMILLYISNPS
            :* ***:.  ** **::* .**.*:******:**:******  :** **:*::* :*.**.

Human       SIILAVTAANTDMATSEALKISREVDPDGRRTLAVITKLDLMDAGTDAMDVLMGRVIPVK 238
Mouse       SIILAVTAANTDMATSEALKISREVDPDGRRTLAVITKLDLMDAGTDAMDVLMGRVIPVK
Frog        SIILAVTAANTDMATSEALKIARESDPDGRRTLAVITKLDLMDAGTDAMDVLLGRVIPVK
Zebrafish   SIILAVTAANTDMATSEALKVAREVDPDGRRTLAVVTKLDLMDAGTDAMDVLMGRVIPVK
Fruitfly    SIILAVTAANTDMATSEALKLAKDVDPDGRRTLAVVTKLDLMDAGTDAIDILCGRVIPVK
Nematode    SIILAVTPANQDFATSEPIKLAREVDAGGQRTLAVLTKLDLMDQGTDAMDVLMGKVIPVK
            ******* ** *:**** :*:::: * .*:*****:******* ****:*:* *:*****

Human       LGIIGVVNRSQLDINNKKSVTDSIRDEYAFLQKKYPSLANRNGTKYLARTLNRLLMHHIR 298
Mouse       LGIIGVVNRSQLDINNKKSVTDSIRDEYAFLQKKYPSLANRNGTKYLARTLNRLLMHHIR
Frog        LGIIGVVNRSQLDINNKKIVADSIRDEYGFLQKKYPSLATRNGTKYLARTLNRLLMHHIR
Zebrafish   LGLIGVVNRSQLDINNKKSVADSIRDEHGFLQKKYPSLANRNGTKYLARTLNRLLMHHIR
Fruitfly    LGIIGVMNRSQKDIMDQKHIDDQMKDEAAFLQRKYPTLATRNGTPYLAKTLNRLLMHHIR
Nematode    LGIIGVVNRSQQNILDNKLIVDAVKDEQSFMQKKYPTLASRNGTPYLAKRLNMLLMHHIR
            **:***:**** :* ::* : * ::** .*:*:***:**.**** ***: ** *******
               BSE (connecting helix)                        L1N (IDR)
Human       DCLPELKTRINVLAAQYQSLLNSYGEPVDDKSATLLQLITKFATEYCNTIEGTAKYIETS 358
Mouse       DCLPELKTRINVLAAQYQSLLNSYGEPVDDKSATLLQLITKFATEYCNTIEGTAKYIETS
Frog        DCLPELKTRINVLAAQYQTLLNSYGEPVEDKSATLLQLITKFATEYCNTIEGTAKYIETS
Zebrafish   DCLPELKTRINVLSAQYQSLLSSYGEPVEDMSATLLQLITKFATEYCNTIEGTAKYIETA
Fruitfly    DCLPDLKTRVNIMATQFQSLLNSYGEDVSDKSQTLLQIITKFSSAYCCTIEGTARNIETT
Nematode    NCLPALKARVSIMNAQCQSDLVAFGEPVEDKNRTLLQIITRFATAYTSTIEGTARNIETT
            :*** **:*:.:: :* *: * :*** *.* . ****:**:*:: *  ******: ***:

Human       ELCGGARICYIFHETFGRTLESVDPLGGLNTIDILTAIRNATGPRPALFVPEVSFELLVK 418
Mouse       ELCGGARICYIFHETFGRTLESVDPLGGLNTIDILTAIRNATGPRPALFVPEVSFELLVK
Frog        ELCGGARICYIFHETFGRTLESVDPLGGLTTIDVLTAIRNATGPRPALFVPEVSFELLVK
Zebrafish   ELCGGARICYIFHETFGRTLESVDPLGGLTTIDVLTAIRNATGPRPALFVPEVSFELLVK
Fruitfly    ELCGGARMGYIFHETFGRTLDSIHPLAGLSKMDILTAIRNATGPRPALFVPEVSFELLVK
Nematode    ELCGGARICYIFHDTFGRSLESVNPLENLTQLDILTAIRNATGPRPALFVPEVSFELLVK
            *******: ****:****:*:*:.** .*. :*:**************************

Human       RQIKRLEEPSLRCVELVHEEMQRIIQHCSNYSTQELLRFPKLHDAIVEVVTCLLRKRLPV 478
Mouse       RQIKRLEEPSLRCVELVHEEMQRIIQHCSNYSTQELLRFPKLHDAIVEVVTCLLRKRLPV
Frog        RQVKRLEEPSLRCVELVHEEMQRIIQHCSNYSTQELLRFPKLHDAIVEVVTSLLRKRLPV
Zebrafish   RQVKRLEEPSLRCVELVHEEMQRIIQHCSNYSTQELLRFPKLHDAIVEVVTSLLRKRLPV
Fruitfly    RQIRRLEEPSLRCVELIHEEMQRIVQHCGNEVQQEMLRFPKLHEKIVDVVTQLLRRRLPH
Nematode    RQIQRLEEPSLRCVELVHEEMQRMVQHCGFTTQQEMIRFPRLYDKINEVVSGVLKERLKP
            **::***********:******::***.    **::***:*:: * :**: :*:.**

Human       TNEMVHNLVAIELAYINTKHPDFADACGLMNNNIEEQRRNRLAREL---PSAVSRDKVA- 534
Mouse       TNEMVHNLVAIELAYINTKHPDFADACGLMNNNIEEQRRNRLAREL---PSAGSRDKVP-
Frog        TNEMVHNLVAIELAYINTKHPDFADACGLINNNIEEQRRNRISRDL---PPPAASRDKA-
Zebrafish   TNEMVHNLVAIELAYINTKHPDFADACGLMNNNIEEQRRNRM-REL---PTSVPRDKMA-
Fruitfly    TNVMVENIVAIELAYINTKHPDFHKDAALVPSLLKTDSDPYSQINLGQRRANTPRNHMSP
Nematode    TNELVENLVAIELAYINTKHPEFTEANLVTLLKEELLLDDRHGRSRNRHASTGERAQLQP
            ** :*.*:*************:* .    :     :           .            .
```

```
human       ---SGGGGVGDG-VQ--------------------------EPTTGNWRGMLKTSKAEEL 564
Mouse       ---SAGGGIGDG-GQ--------------------------EPTTGNWRGMLKTSKAEEL
Frog        ---APGSSTGDG-LP--------------------------DAGTGNWRGMMKAKG-EEA
Zebrafish   ---GGA----QA-EQ--------------------------EGGTGTWRGMLKKG--DEG
Fruitfly    QISSHSAGSQQPQQQQPPQPNSSQQQYSQVHEQNHVAENSTPSMASTWLSNILPPAPTRP
Nematode    VPGVNGVDLNAVLQQQ--------Q----QQSQN-------QRASNGFLGLFGNA-----
                 .                                      :. :  . :

human       ---LAEEKSKPIPIMPASPQKGHAVNL-LDVPV-PVARKLSAREQRDCEVIERLIKSYFL 619
Mouse       ---LAEEKSKPIPIMPASPQKGHAVNL-LDVPV-PVARKLSAREQRDCEVIERLIKSYFL
Frog        ---SVEEKNKAPPAFPASPLRGHAVNL-LDVPV-PVARKLSAREQRDCEVIERLIKSYFL
Zebrafish   ---QGEEKTKLQSSIPASPQKGHAVNL-LDVPV-PVARKLSAREQRDCEVIERLIKSYFL
Fruitfly    DSIENSTNNTPVHNNIVSPV--KPVNLLPDVPANHNPRRLTDKEQKDCDVIEHLIKSYFY
Nematode    -----------AASSKTSPQEKQSANFLPEVPETQLGRKLTSREQRDVAIIERLIRNYFI
                            .**    : .*:  :**      *:*: :**:*  :**:**:.**

                                                          BSE (C-term helix)
human       IVRKNIQDSVPKAVMHFLVNHVKDTLQSELVGQLYKSSLLDDLLTESEDMAQRRKEAADM 679
Mouse       IVRKNIQDSVPKAVMHFLVNHVKDTLQSELVGQLYKSSLLDDLLTESEDMAQRRKEAADM
Frog        IVRKNIQDSVPKAVMHFLVNHVKDTLQSELVGQLYKSLLLEDLLTESEDMAQRRNEAADM
Zebrafish   IVRKNIQDSVPKAVMHFLVNHVKDSLQSELVGQLYKPALLDDLLTESEDMAQRRNEAADM
Fruitfly    IVRKSIQDSVPKAIMHFLVNYVKDNLQSELVTHLYKSDKAETLLNESDHIAVRRKEAADM
Nematode    IVRKNIQDSVPKAIMALLVNFVRDNLQSELVRQLYKPDEMDDLLAETEDMAQRRRDTLET
            ****.********:* :***.*:*.****** :***    : ** *::.:* **.:: :

                          CT-SLiM
human       LKALQGASQIIAEIRETHLW 699
Mouse       LKALQGASQIIAEIRETHLW
Frog        LKALQRASQIIAEIRETHLW
Zebrafish   LKALQKASQVIAEIRETHLW
Fruitfly    LKALTRANHIISEIRETHMW
Nematode    MKALQQASVIISEVRETQVW
            :***  *. :*:*:***::*
```

Variable domain (VD)/IDR

GTPase effector domain (Stalk)

*** Identical, fully conserved   : Strongly similar   . Weakly similar**

B

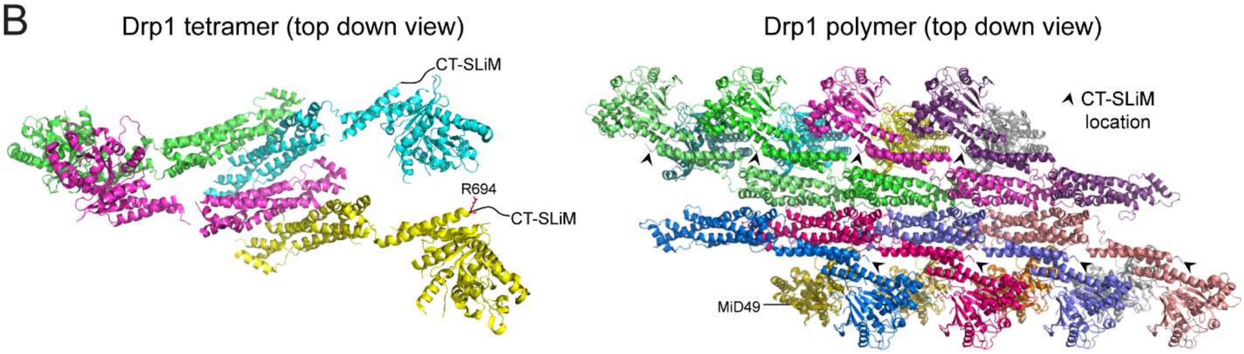

C

	Drp1 variant	CT-SLiM truncation or extension
	WT	^{694}RETHLW^{699}
truncated	ΔCT4	^{694}RE^{695}
truncated	ΔCT6	-
extended	CT+	^{694}RETHLW^{699} + ADPLEVLFQGPKLAAALEHHHHHH (24)
extended	CT+^{sh}	^{694}RETHLW^{699} + ADPLEVLFQ (9)
extended	CT+*	^{694}RETHLW^{699} + GIGSGSCCPGCCGG (14)

Figure S2

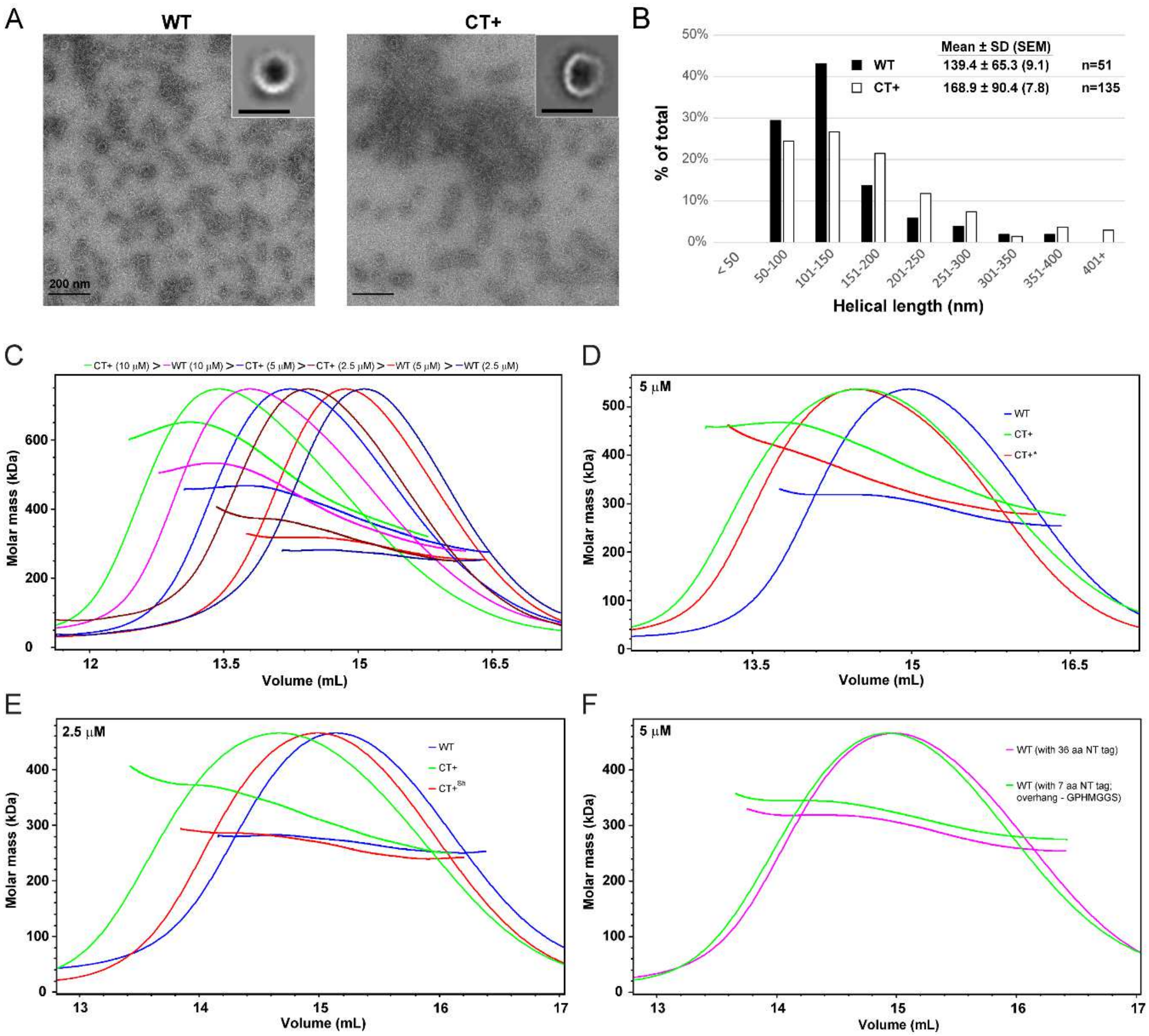

A
WT
CT+
200 nm
B
Mean ± SD (SEM)
WT 139.4 ± 65.3 (9.1) n=51
CT+ 168.9 ± 90.4 (7.8) n=135
% of total
Helical length (nm)
< 50
50-100
101-150
151-200
201-250
251-300
301-350
351-400
401+
C
CT+ (10 μM) > WT (10 μM) > CT+ (5 μM) > CT+ (2.5 μM) > WT (5 μM) > WT (2.5 μM)
Molar mass (kDa)
Volume (mL)
D
5 μM
WT
CT+
CT+*
E
2.5 μM
WT
CT+
F
5 μM
WT (with 36 aa NT tag)
WT (with 7 aa NT tag; overhang - GPHMGGS)

Figure S3

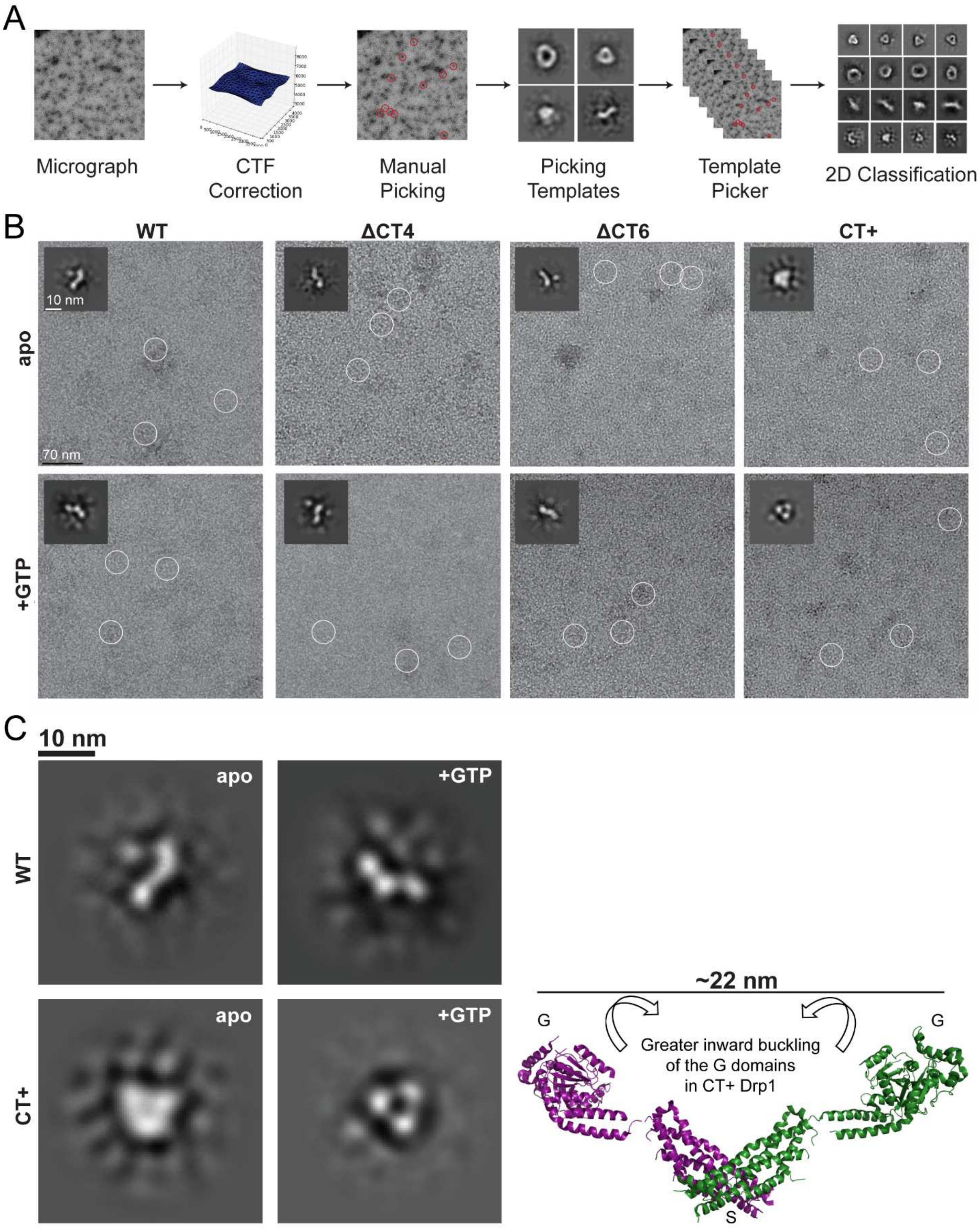
A
Micrograph
CTF Correction
Manual Picking
Picking Templates
Template Picker
2D Classification
B
WT
ΔCT4
ΔCT6
CT+
apo
+GTP
10 nm
70 nm
C
10 nm
apo
+GTP
WT
CT+
~22 nm
G
G
Greater inward buckling of the G domains in CT+ Drp1
S

Figure S4

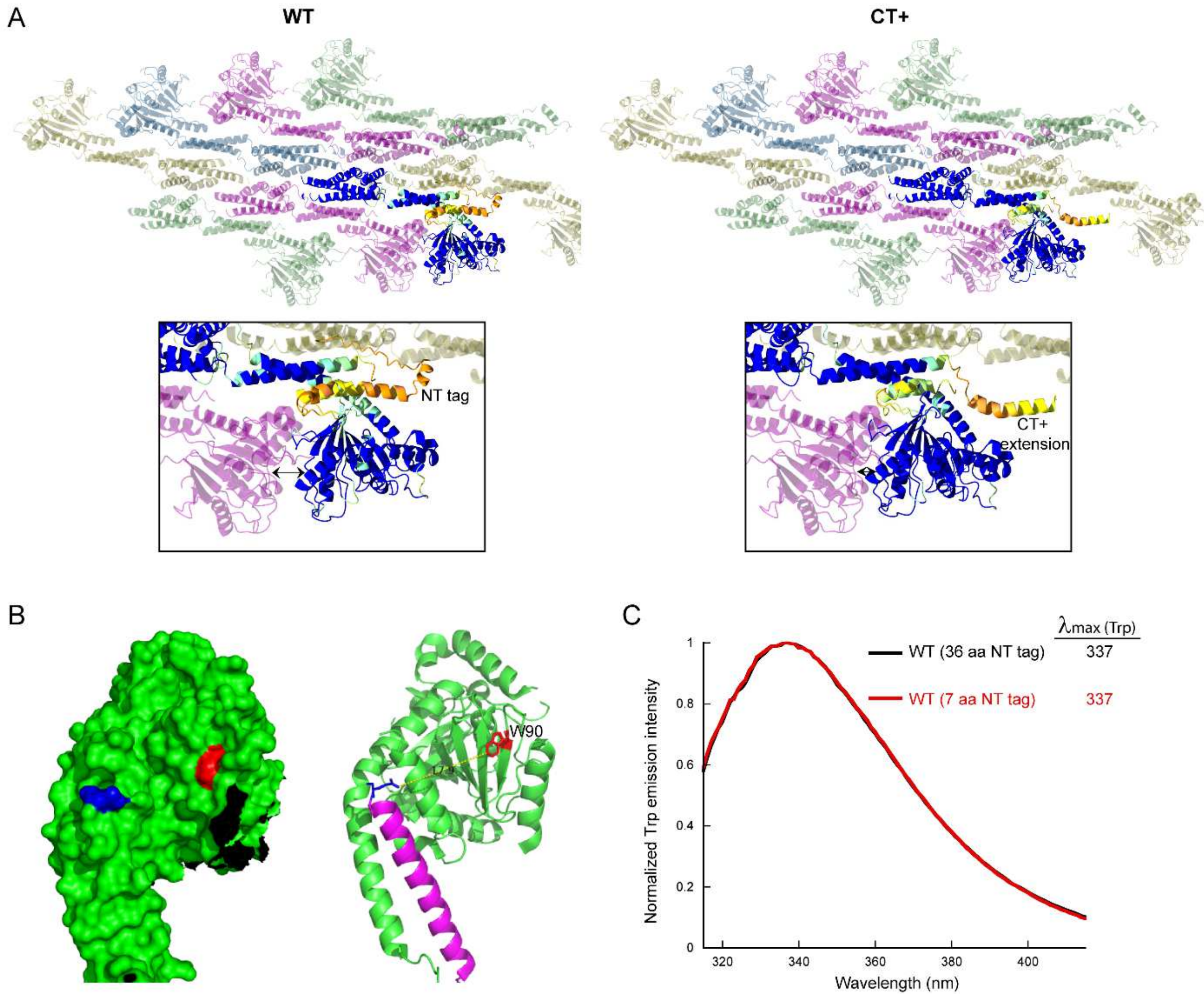
A
WT
CT+
NT tag
CT+
extension
B
W90
C
λmax (Trp)
WT (36 aa NT tag) 337
WT (7 aa NT tag) 337
Normalized Trp emission intensity
Wavelength (nm)
0
0.2
0.4
0.6
0.8
1
320
340
360
380
400

Figure S5

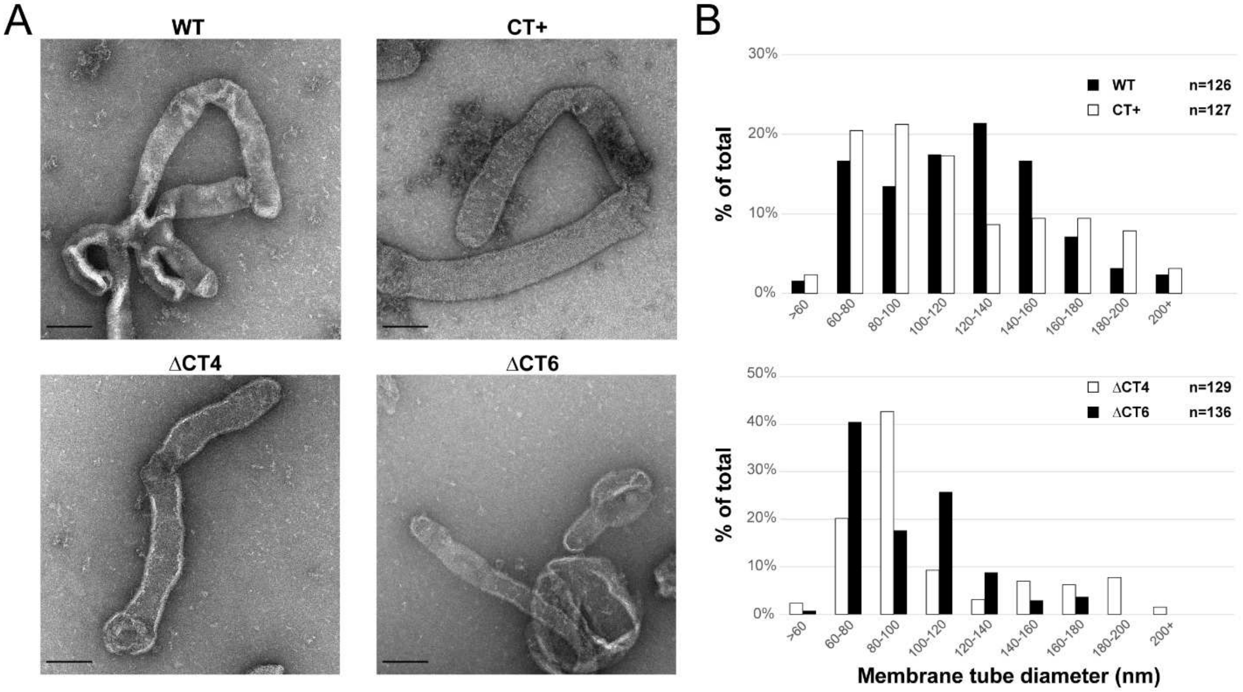
A
WT
CT+
ΔCT4
ΔCT6
B
30%
20%
10%
0%
% of total
WT n=126
CT+ n=127
>60
60-80
80-100
100-120
120-140
140-160
160-180
180-200
200+
50%
40%
30%
20%
10%
0%
ΔCT4 n=129
ΔCT6 n=136
Membrane tube diameter (nm)

Figure S6

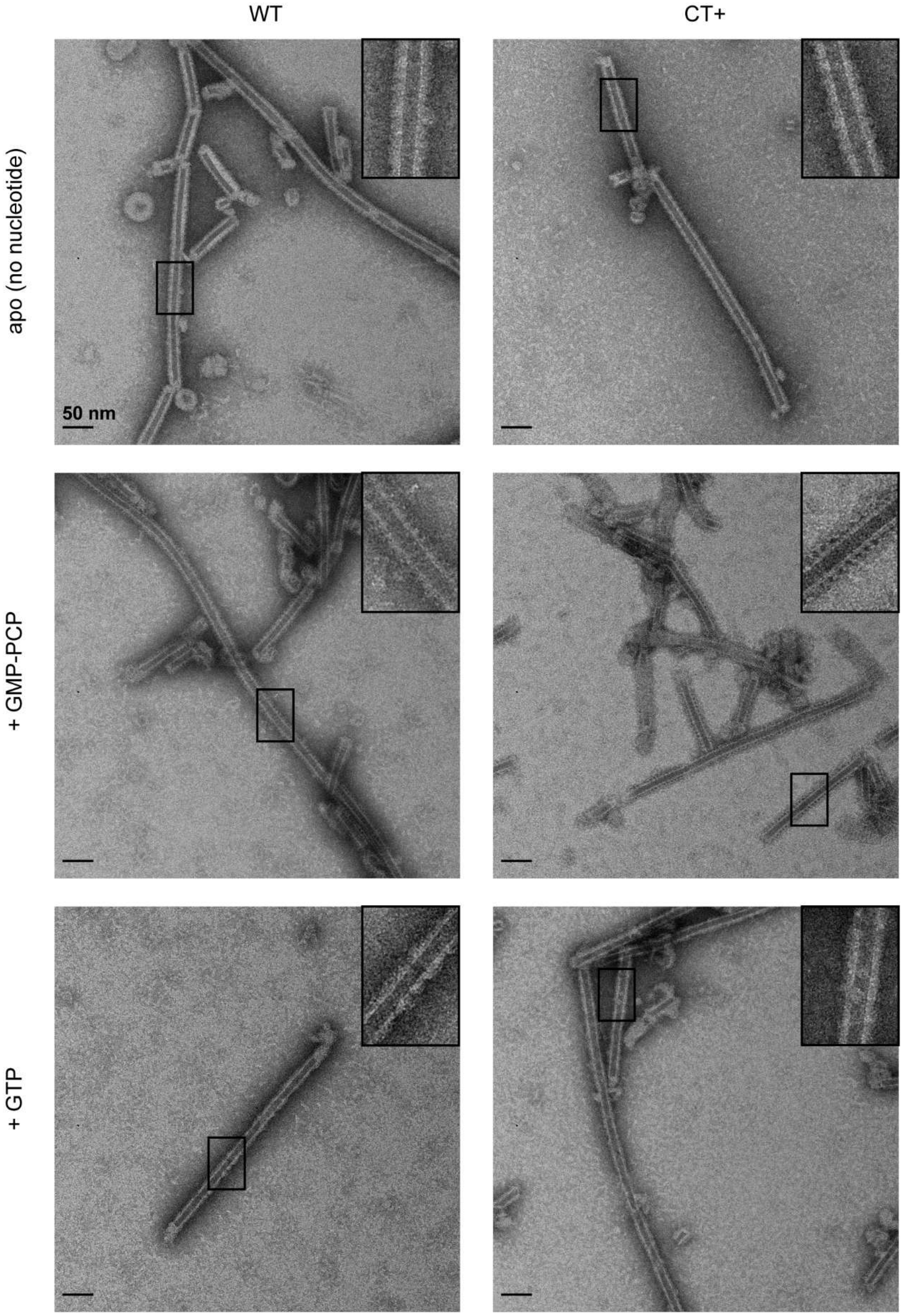

Figure S7

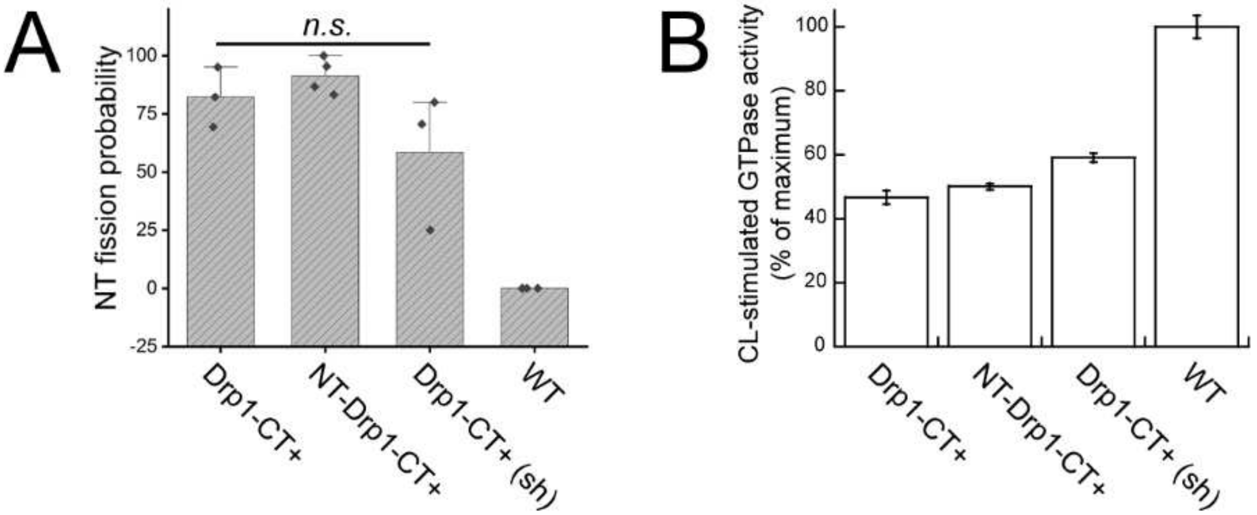
A
n.s.
NT fission probability
100
75
50
25
0
-25
Drp1-CT+
NT-Drp1-CT+
Drp1-CT+ (sh)
WT
B
CL-stimulated GTPase activity
(% of maximum)
100
80
60
40
20
0
Drp1-CT+
NT-Drp1-CT+
Drp1-CT+ (sh)
WT

Figure S8

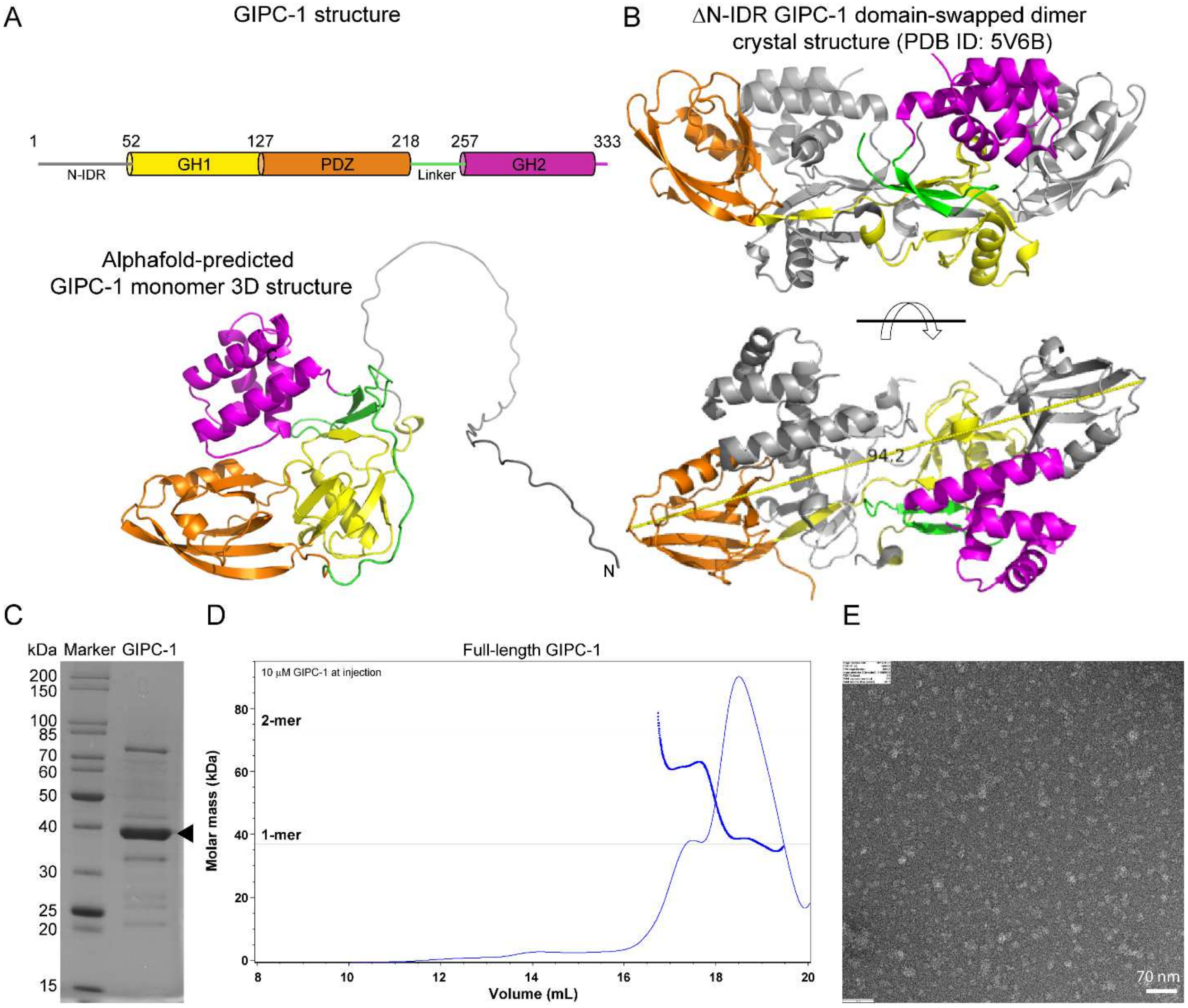

A
GIPC-1 structure
1
52
127
218
257
333
N-IDR
GH1
PDZ
Linker
GH2
Alphafold-predicted
GIPC-1 monomer 3D structure
N
B
ΔN-IDR GIPC-1 domain-swapped dimer
crystal structure (PDB ID: 5V6B)
94.2
C
kDa Marker GIPC-1
200
150
100
85
70
60
50
40
30
25
20
15
D
Full-length GIPC-1
10 μM GIPC-1 at injection
2-mer
1-mer
Molar mass (kDa)
Volume (mL)
E
70 nm

Figure S9

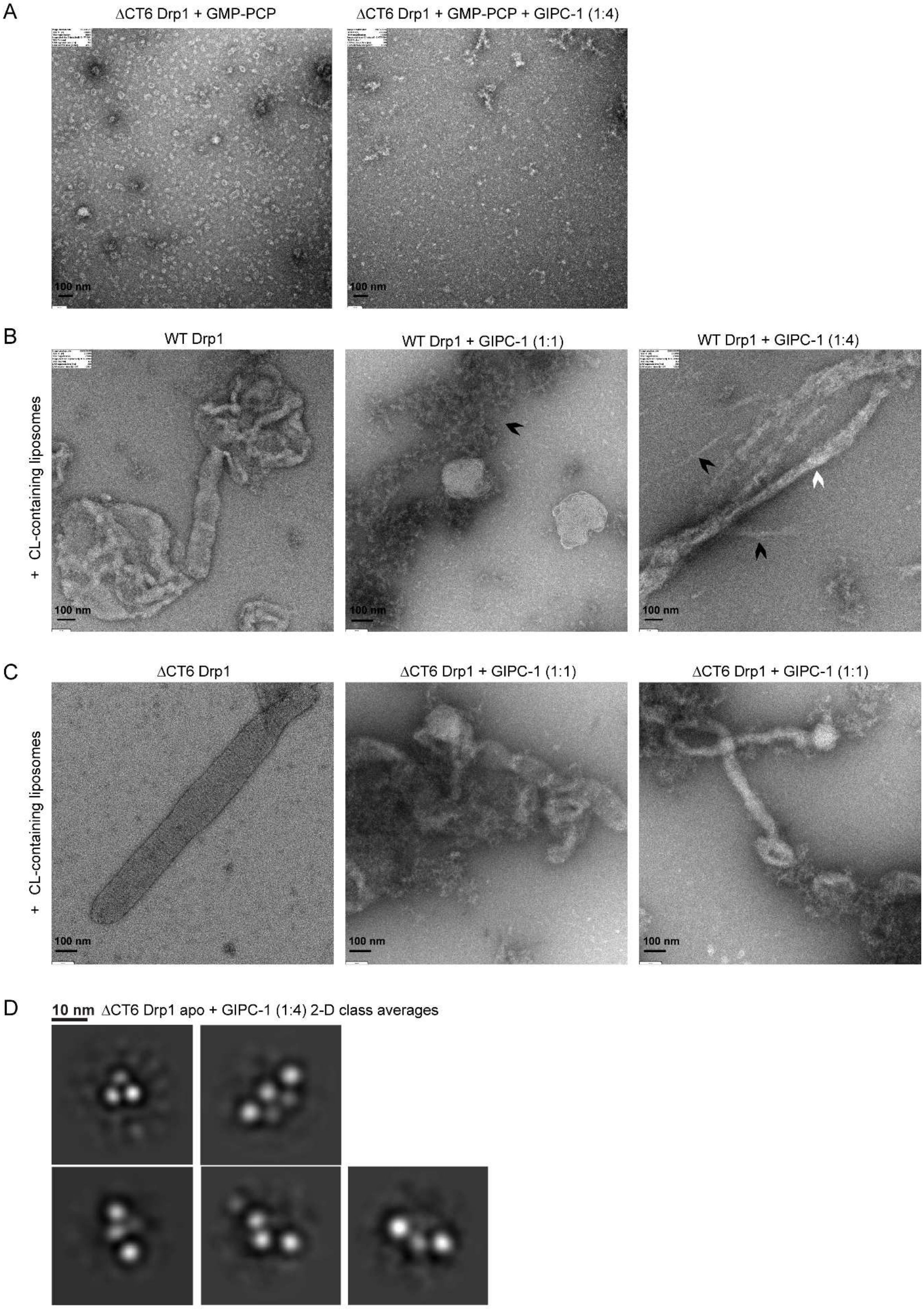
A
ΔCT6 Drp1 + GMP-PCP
ΔCT6 Drp1 + GMP-PCP + GIPC-1 (1:4)
100 nm
100 nm
B
WT Drp1
WT Drp1 + GIPC-1 (1:1)
WT Drp1 + GIPC-1 (1:4)
+ CL-containing liposomes
100 nm
100 nm
100 nm
C
ΔCT6 Drp1
ΔCT6 Drp1 + GIPC-1 (1:1)
ΔCT6 Drp1 + GIPC-1 (1:1)
+ CL-containing liposomes
100 nm
100 nm
100 nm
D
10 nm
ΔCT6 Drp1 apo + GIPC-1 (1:4) 2-D class averages

Figure S10

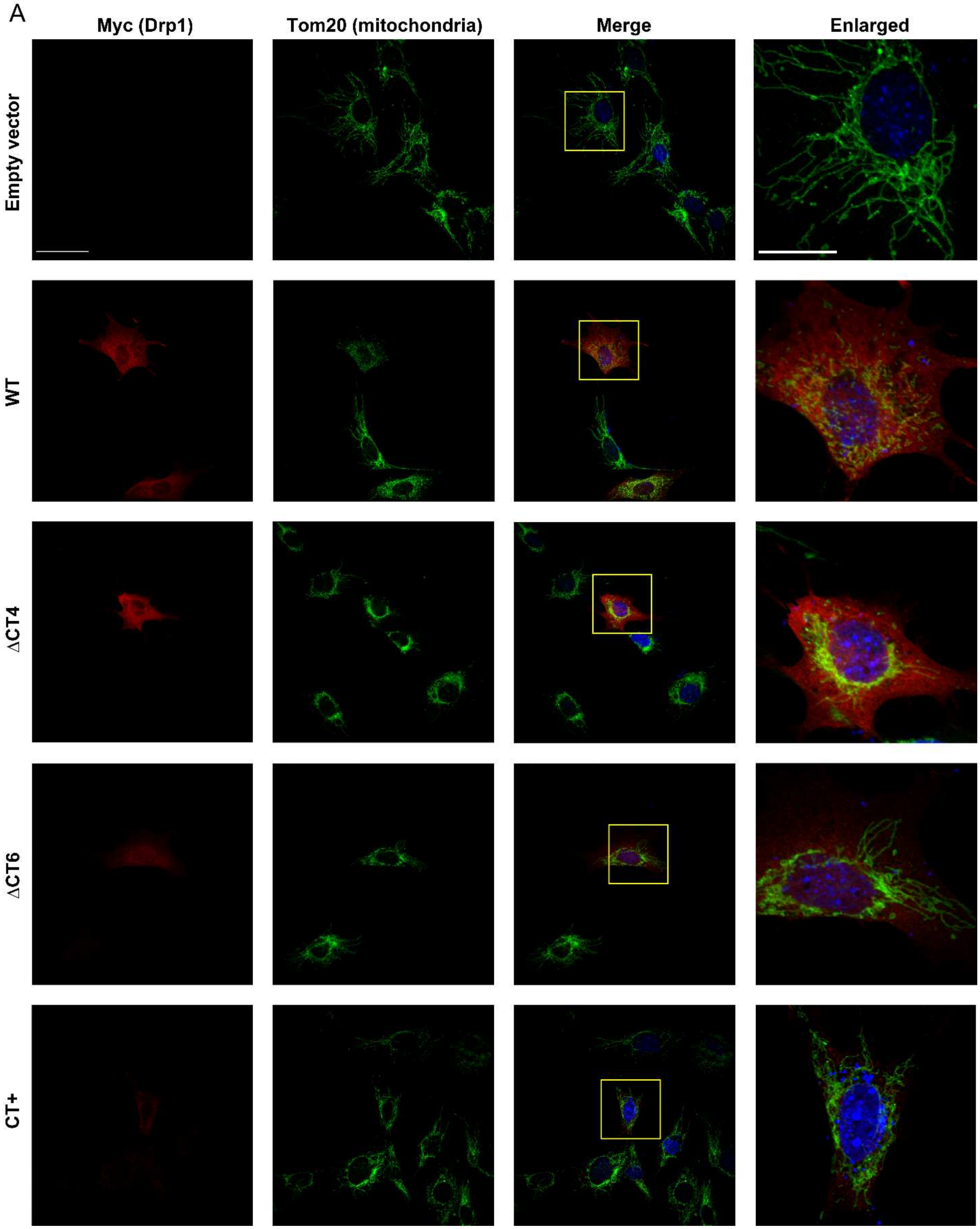
A
Myc (Drp1)
Tom20 (mitochondria)
Merge
Enlarged
Empty vector
WT
ΔCT4
ΔCT6
CT+

Figure S11

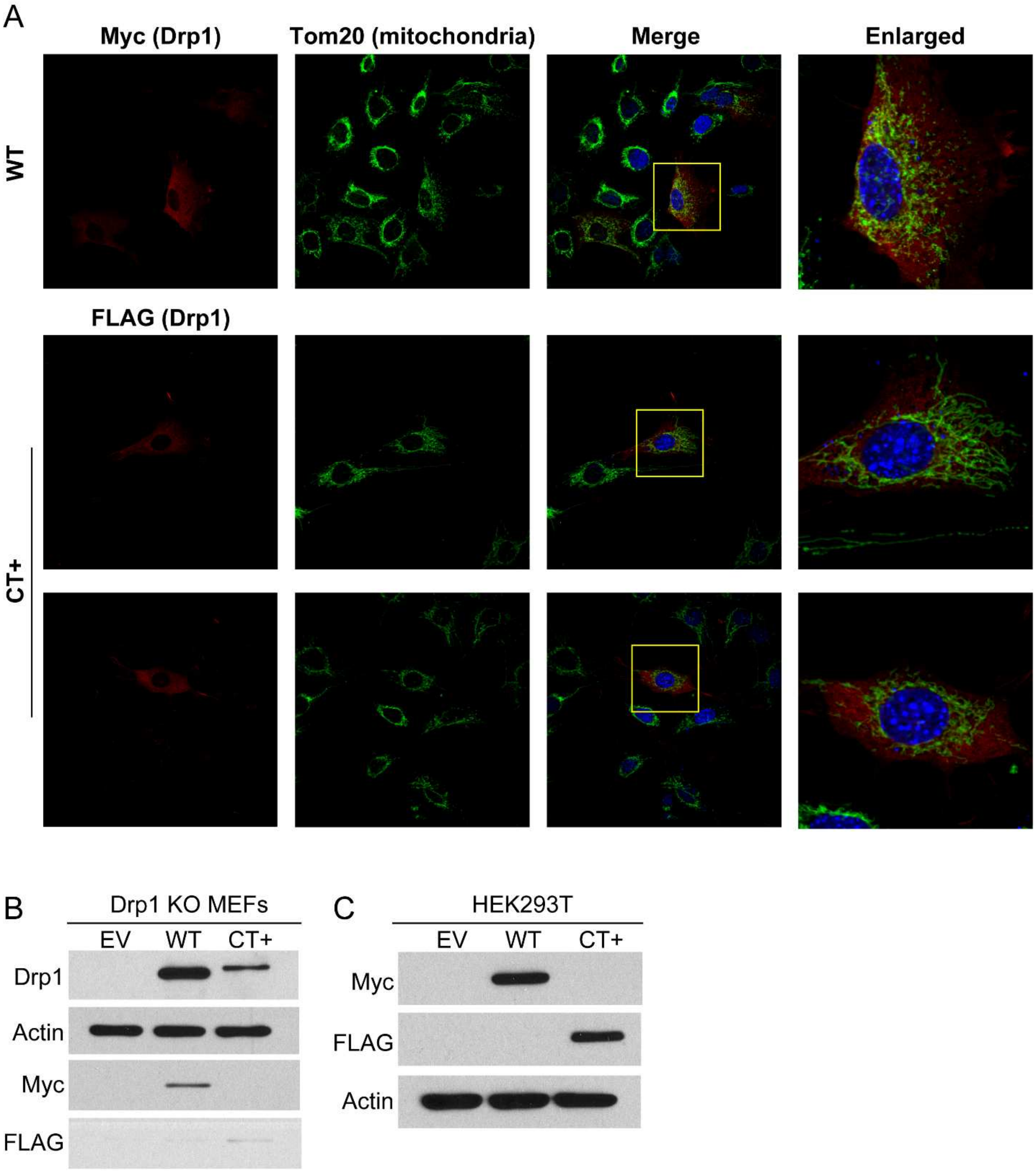
A
Myc (Drp1)
Tom20 (mitochondria)
Merge
Enlarged
WT
FLAG (Drp1)
CT+
B
Drp1 KO MEFs
EV
WT
CT+
Drp1
Actin
Myc
FLAG
C
HEK293T
EV
WT
CT+
Myc
FLAG
Actin

Supplementary Files

This is a list of supplementary files associated with this preprint. Click to download.

- SupplementaryMovie1.avi
- SupplementaryMovie2.avi
- SupplementaryMovie2.avi
- SupplementaryMovie4.avi
- SupplementaryMovie5.avi
